# Recent Advances in DNA Nanotechnology-Enabled Biosensors for Virus Detection

**DOI:** 10.3390/bios13080822

**Published:** 2023-08-15

**Authors:** Lihui Yuwen, Shifeng Zhang, Jie Chao

**Affiliations:** 1 State Key Laboratory of Organic Electronics and Information Displays, Jiangsu Key Laboratory for Biosensors, Institute of Advanced Materials (IAM), Nanjing University of Posts and Telecommunications, Nanjing 210023, China; iamlhyuwen@njupt.edu.cn (L.Y.); iamsfzhang1257@gmail.com (S.Z.); 2 School of Geography and Biological Information, Nanjing University of Posts and Telecommunications, Nanjing 210023, China

**Keywords:** DNA nanotechnology, biosensors, DNA origami, DNA tile, virus detection

## Abstract

Virus-related infectious diseases are serious threats to humans, which makes virus detection of great importance. Traditional virus-detection methods usually suffer from low sensitivity and specificity, are time-consuming, have a high cost, etc. Recently, DNA biosensors based on DNA nanotechnology have shown great potential in virus detection. DNA nanotechnology, specifically DNA tiles and DNA aptamers, has achieved atomic precision in nanostructure construction. Exploiting the programmable nature of DNA nanostructures, researchers have developed DNA nanobiosensors that outperform traditional virus-detection methods. This paper reviews the history of DNA tiles and DNA aptamers, and it briefly describes the Baltimore classification of virology. Moreover, the advance of virus detection by using DNA nanobiosensors is discussed in detail and compared with traditional virus-detection methods. Finally, challenges faced by DNA nanobiosensors in virus detection are summarized, and a perspective on the future development of DNA nanobiosensors in virus detection is also provided.

## 1. Introduction

Virus-related infectious diseases greatly threaten human beings all over the world. The outbreak of COVID-19 [1,2], caused by SARS-CoV-2, has further highlighted the importance of preventing and controlling virus diseases. The detection of viruses with high sensitivity in a short time is essential to eliminate the source of infection and prevent the transmission of the disease. Currently, virus detection primarily relies on nucleic acid detection [3,4,5,6,7] and protein detection [8,9,10]. The nucleic acid-detection strategy for viruses involves the detection of viral DNA or RNA, which has the merit of high sensitivity and specificity but can be time-consuming and costly. Commonly used methods for nucleic acid detection include real-time quantitative polymerase chain reaction (RT–PCR) [4], loop-mediated isothermal amplification (LAMP) [3], and recombinase polymerase amplification (RPA) [11]. The protein-detection strategy for viruses includes antigen detection and antibody detection. Antigen detection targets viral surface antigens, while antibody detection utilizes the corresponding antibodies produced by the host immune system toward viral antigens. Protein assays are advantageous for their fast and cost-effective detection, but they may have lower sensitivity and specificity. Commonly used protein-detection methods include a serum-neutralization test, an enzyme-linked immunosorbent assay (ELISA) [6], an immunocolloidal gold test, and an indirect immunofluorescence technique (IF) [12].

The DNA molecule is garnering significant interest as a scaffold material to construct biosensors. The versatility of DNA structures allows for the detection of numerous biological targets, including nucleic acids, proteins, metal ions, and small molecules. Compared to commonly used biosensors, DNA-based biosensors offer significant advantages, including excellent bioactivity, facile addressability, structural designability, etc. For instance, DNA aptamers, obtained through artificial screening and modification, exhibit excellent thermal stability [13,14,15], bioaffinity [16,17,18], and stability [19]. Moreover, DNA molecules can construct programmable supramolecular nanostructures and serve as templates for achieving the precise control of sensing elements over the spatial arrangement, resulting in a remarkable improvement in sensing performance and enabling innovative biosensors [20,21,22,23,24,25,26]. With the remarkable progress in DNA nanotechnology, dynamic networks constructed through DNA hybridization can adeptly amplify biosensing signals, making DNA biosensors more effective for virus detection. DNA nanotechnology-based virus biosensors have high sensitivity and specificity, fast response, simple operation, short detection time, etc., which make them highly promising for virus detection [27,28,29].

**Scheme 1:**
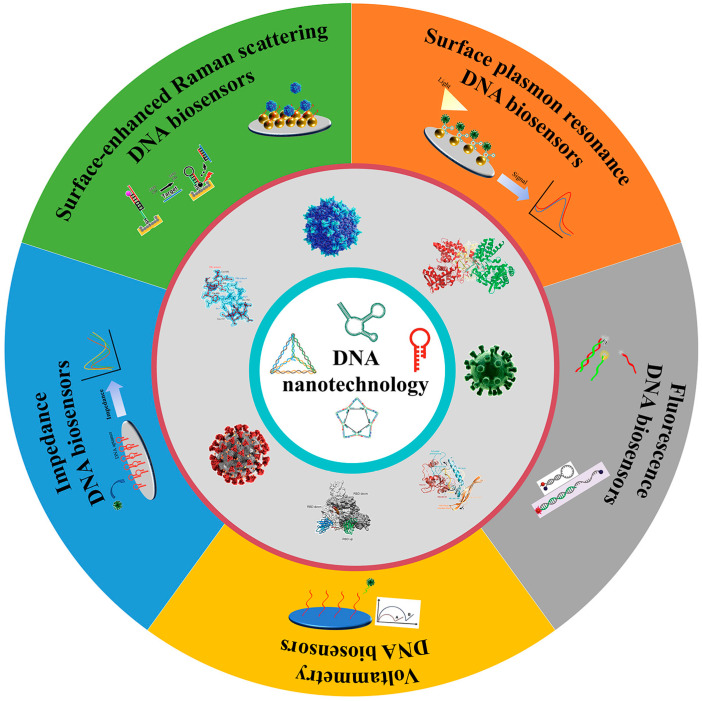
DNA nanotechnology-based optical and electrochemical DNA biosensors for virus detection.

In recent years, biosensors based on DNA nanotechnology have gained increasing attention due to their unique properties and great potential for virus detection [30,31]. Many types of DNA nanobiosensors have been developed using electrical, optical, acoustic, and magnetic-sensing technologies [32,33]. In comparison, optical and electrochemical DNA nanobiosensors exhibit high sensitivity, wide linear range, low cost, easy operation, short detection time, high stability to the environment, etc. Although many research works on DNA nanobiosensors have been reported [34,35,36], there remains a necessity for a comprehensive elucidation of recent advances in DNA nanobiosensors for virus detection. This article provides a brief introduction to DNA nanotechnology, focusing on DNA tile and DNA aptamer (Scheme 1). Then, structural features and the classification of viruses, followed by an overview of traditional nucleic acid and protein-detection methods for viruses. Then, the state-of-the-art advance for optical and electrochemical DNA biosensors for virus detection based on DNA nanotechnology is discussed in detail. This review may serve as a valuable reference for researchers to develop more effective and sensitive virus-detection methods and sensors in the field of biomedicine.

## 2. DNA Nanotechnology

DNA nanotechnology, developed by Nadrian Seeman in 1982 [37], has undergone more than 40 years of rigorous development and has been used in a variety of fields, particularly in chemistry, material science, and biomedicine [38,39,40,41,42]. As commonly used DNA nanotechnology in DNA biosensors, DNA tiles and DNA aptamers are briefly introduced.

### 2.1. DNA Tile

**Figure 1:**
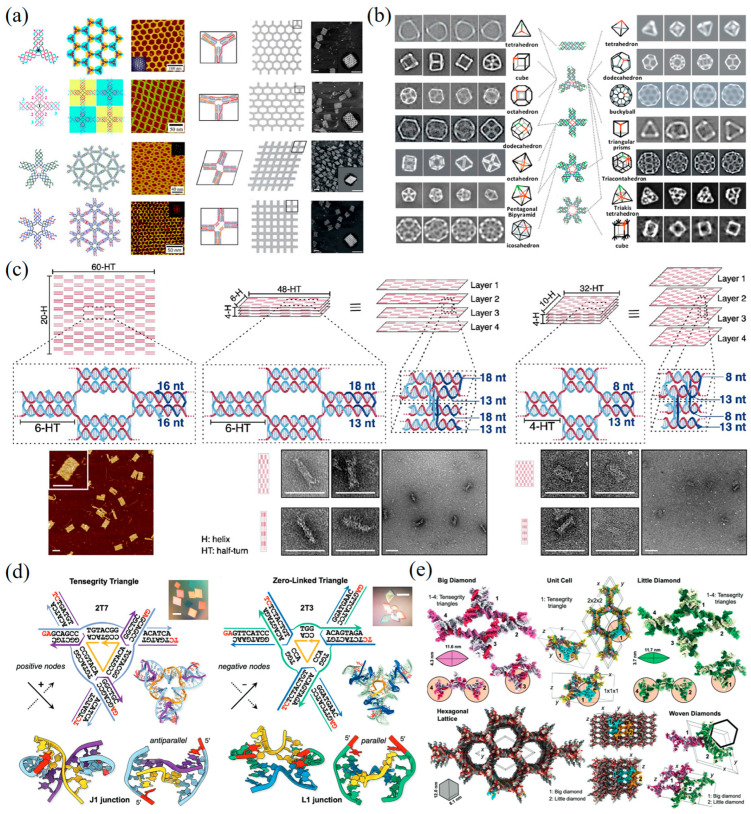
Wireframe DNA tile self-assembled polyhedron. (**a**) The infinite 2D lattice is constructed separately using the multi-vertex branching DNA tile as the basic unit [43,45,46,47,48]. Copyright 2020, American Chemical Society. (**b**) Construction of wireframe polyhedral structures by hierarchical assembly of branching DNA tiles as basic units [48,49,50,51,52,53,54,55,56]. Copyright 2020, American Chemical Society. (**c**) Two-dimensional (2D) and three-dimensional (3D) lattices of composite 142 mesojunctions [57]. Copyright 2023, American Chemical Society. (**d**) 3D DNA motifs with two turns per helical axis [58]. Copyright 2022, Wiley-VCH. (**e**) Crystal packing of the sΔ247-WC 24 bp trigonal motif with GA/TC sticky ends showcasing the two diamond subunits [59]. Copyright 2022, Wiley-VCH.

DNA tile is a highly sophisticated self-assembly technique that relies exclusively on short ssDNA chains. The process involves organizing ssDNA hangers into unit modules, followed by the design and construction of a two-dimensional (2D) array of DNA using star-shaped branched DNA, resulting in a highly ordered finite structure (Figure 1a) [43]. The individual addressing of each DNA chain in DNA tile-unit blocks allows for precise control over the final structure [44]. This innovative technology has been explored within functional nanomaterials that can be used in a variety of applications.

The first tile structure in DNA nanotechnology was the fixed “Holiday junction” [37,60]. Since then, Seeman et al. have designed and synthesized more stable DNA tile structures with 3-arm [61], 6-arm [62], 8-arm [63], and 12-arm [63], as well as modules such as DX (double crossover) [64], TX (triple crossover) [65], PX (paranemic crossover) [66], and 6 HB (six-helix bundles) [67]. Researchers have also constructed multi-dimensional structures using DNA tile modules. Mao et al. utilized three-point-star, four-point-star, and six-point-star structures as basic units to create 2D hexagonal, quadrilateral, and triangular DNA lattice structures (Figure 1a,b) [45,46,47,48,49,50,51,52,53,54,55,56]. Wei et al. improved the traditional DNA tile module structure by using 3-arm or 4-arm mixed crossover tiles as basic units, which were assembled in one-dimensional (1D) and 2D periodic arrays through complementary base pairing between sticky ends, using both scaffold-free and scaffolded methods (Figure 1c) [57].

DNA polyhedra using DNA tile modules can yield specific structures by adjusting the length and curvature of DNA branches, which allows for the connection of sticky ends between branches to form desired structures [68]. These well-formed DNA tile polyhedra can be used to construct 3D DNA crystals, breaking the nanoscale limit and allowing DNA tile structures to be sized to the micron scale in 3D space. For example, 3-arm tile units have been used to construct structures such as tetrahedrons [69] and octahedrons [70]. Yan et al. used the layered-crossover motif to construct 3D-framework DNA origami structures, using a set of diamond-shaped layered crossover DNA tiles with accurately adjustable angles to assemble into 3D crystals up to several hundred microns in size [71]. Mao et al. found that 3D DNA patterns with 24 bp repeating edges and non-integer transitions can produce controlled macroscopic self-assembly in 3D (Figure 1d,e) [58,59]. The pattern transcends the integer transitions paradigm, and the “rule of thirds” approach opens the door for exploring the topological self-assembly in designed nanomaterials.

DNA nanostructures built using DNA tile can be designed as stable 1D, 2D, or 3D platforms [72], precisely controlling the spacing, valency, and spatial arrangement of ligands, which can facilitate the design of novel biosensors [31].

### 2.2. DNA Aptamer

**Figure 2:**
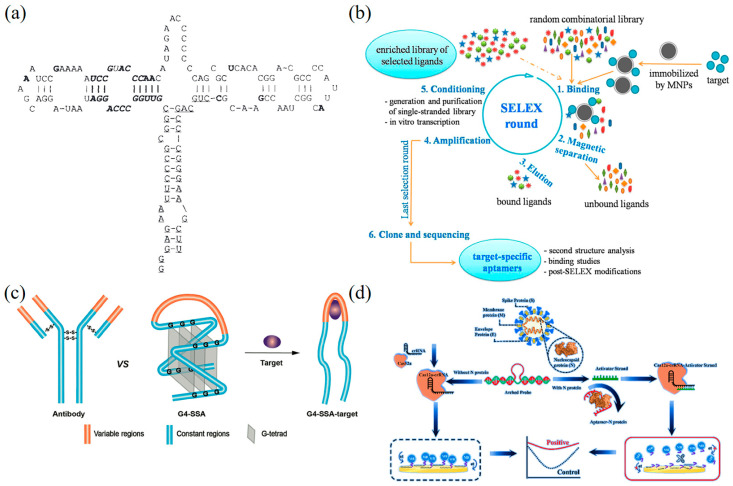
Principles and applications of DNA aptamers for biosensing. (**a**) Secondary structure of the B4–25 aptamer obtained by the FOLD method [74]. Copyright 1990, Springer Nature. (**b**) The SELEX process consists of five main steps: binding, magnetic separation, elution, amplification, and conditioning [83]. Copyright 2015, American Chemical Society. (**c**) The schematic representation illustrates the structures of the typical antibodies, G4–SSA and G4–SSA target complement [84]. Copyright 2019, American Chemical Society. (**d**) Schematic of the CRISPR/Cas12a-derived electro-chemical sensor for ultrasensitive detection of SARS-CoV-2p [85]. Copyright 2022, Elsevier.

DNA aptamers are a class of oligonucleotide sequences composed of DNA molecules that are screened from nucleic acid libraries by in vitro evolutionary technique (Figure 2a). In 1990, Tuerk and Gold [73] demonstrated a technique for screening RNA sequences from oligonucleotide libraries for specific binding to phage DNA polymerase, namely, the systematic evolution of ligands by the exponential-enrichment (SELEX) technique (Figure 2b). In the same year, Ellington et al. [74] used the SELEX technique to screen for RNA sequences that bind to the dye molecules Cibacron Blue and Reactive Blue 4, and named this oligonucleotide sequence with specific binding ability as an “aptamer”. The development of aptamers as biosensing elements was first conceptualized in the mid-1990s [75]. Afterward, many aptamer-based optical- and electrochemical-sensing platforms were established [76,77,78].

Labeled DNA aptamer sensors can accomplish fluorophore labeling through chemical covalent bonding between the fluorophore and the single-stranded DNA (ssDNA). Typical labeled DNA aptamer probes usually consist of hairpin oligonucleotides labeled with a fluorescent dye and a fluorescent quencher at each end. The stem domain typically consists of 5–8 pairs of complementary nucleotides, whereas the loop domain contains 15–30 nucleotides complementary to the target sequence [79]. In the absence of a target sequence, DNA aptamer probes employ a hairpin structure that brings the fluorescent and quenching moieties in close proximity, resulting in the quenching of the fluorescent signal. The quencher can absorb the fluorescence emitted by the fluorescent moiety. When the probe hybridizes with the target sequence, the loop region forms a rigid double-stranded structure with the target sequence, separating the fluorescent and quenching groups, and the fluorescence emission from the fluorescent dye is restored, indicating the presence of the target sequence [80]. This sensing mechanism allows the labeled DNA aptamer probes to function as an on/off switch, making it a useful tool for sensitive and specific detection of a variety of biochemical analyses [81,82].

While labeled DNA-aptamer biosensors exhibit the advantages of high sensitivity, quantification, and multiplexing capabilities, unlabeled DNA aptamer biosensors can detect the target molecules by directly utilizing structural changes of the DNA aptamer. This approach obviates the necessity for introducing additional markers, thereby reducing external interference and errors [86,87]. The advance of DNA aptamer study has driven the development of various newly designed biosensors, such as the structurally switchable nucleic acid aptamer (SSA) (Figure 2c) [84]. These aptamers induce significant structural changes upon binding to a target molecule, which can be transformed into detectable signals, making SSA a popular choice for biosensing. In addition, molecular beacons have also been widely explored for the design of nucleic acid aptamers [82]. For example, Yamamoto et al. developed a detection technique based on “split” nucleic acid aptamer arrays [88]. Additionally, the efficient trans-cleavage activity and good electrochemical property of Cas12a can be leveraged to create an electrochemical aptasensor based on the CRISPR/Cas12a system. This aptasensor exhibits a broad linear range and a low detection limit, suggesting great potential in the early diagnosis of COVID-19 (Figure 2d). By modulating the surface modification of the nanoparticles and the binding mode of the aptamer, highly specific and sensitive detection of target molecules can be realized. This binding strategy provides new possibilities for the construction of efficient biosensors [83,85,89,90,91,92,93,94].

## 3. Virus Classification Based on Baltimore Classification System

**Figure 3:**
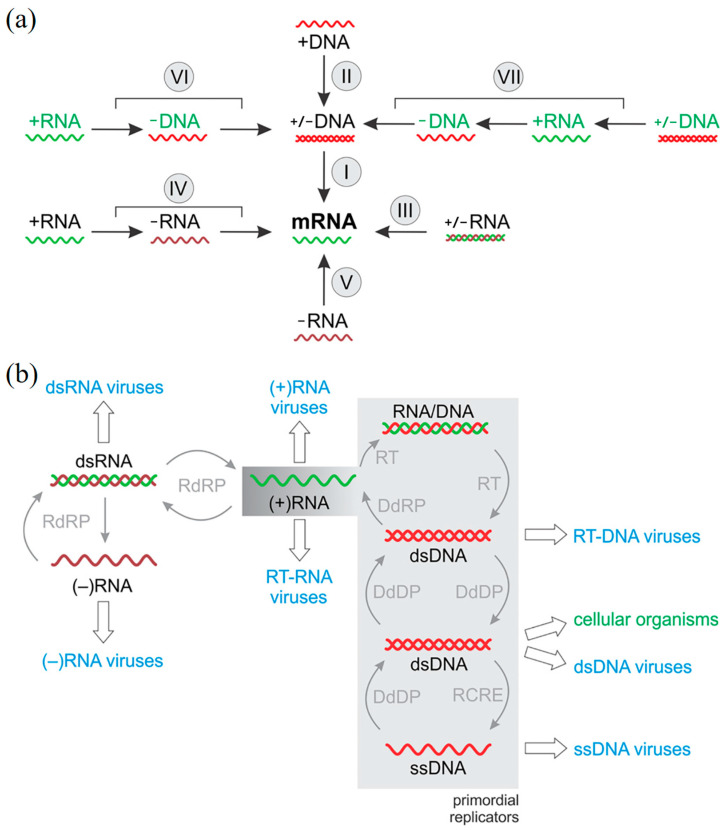
The Baltimore classification of viruses [95]. (**a**) Description of a revised classification scheme for the seven major classes of viruses in the Baltimore classification system that highlights the transfer of genetic information from the nucleic acids of the viral endo-encapsulated genome to the mRNA. (**b**) The evolution of the Baltimore taxa and the original replicon pool is shown. Copyright 2021, American Society for Microbiology.

The Baltimore classification system [95] categorizes viruses into seven groups based on their genome expression pathway (Figure 3a). For the past decades, this system has been a conceptual framework for virology and promotes the development of this field (Figure 3b). This section briefly describes several representative viruses and their molecular characteristics, providing fundamentals for the development of virus DNA biosensors.

### 3.1. Double-Stranded (ds) DNA Viruses

Double-stranded DNA viruses utilize the same classical information transfer pathway as all cells. Adenoviruses, including HAdV, belong to this group of viruses. HAdV was discovered and isolated by Rowe [96] and Hilleman [97] in the 1950s. It is a non-enveloped virus with a diameter of 70–90 nm and contains a linear dsDNA genome ranging from 26–45 kb in size. The hexon, penton, and fiber proteins [98] are the main structural proteins that participate in HAdV serotyping, receptor identification, and infection. Additionally, core proteins like VII and V are involved in virus assembly, maturation, and infection processes [99]. HAdV is divided into seven subgenera (A–G) and has 113 genotypes. The receptor type for different subgenera of HAdV infections varies, mainly depending on the adaptability and diversity of the fiber-protein structure [100,101].

### 3.2. Single-Stranded (ss) DNA Viruses

Single-stranded DNA viruses [95] are a class of viruses with genomes that are composed of a ssDNA molecule, usually including a linear or circular DNA molecule. After infecting host cells, these viruses replicate and reproduce by utilizing the host cell’s organelles and metabolic mechanisms. The genome of ssDNA viruses can be divided into positive and negative strands, and their replication mechanisms are different. During the infection process, the virus utilizes the host cell’s synthesis mechanism to manufacture its proteins and DNA, as well as release new virus particles through the host cell’s division to continue infecting other cells.

AAV is an ssDNA virus belonging to the Parvoviridae family and the Dependoparvovirus subfamily. It was first discovered in the 1960s from laboratory adenovirus preparations and later found in human tissues. AAV has a diameter of approximately 26 nm and is composed of a 20-sided protein shell and a 4.7 kb ssDNA genome. The genome has two inverted terminal repeat sequences (ITRs) at both ends, serving as the virus replication origin and packaging signal. Due to its non-pathogenicity and ability to integrate into the host genome at specific sites, AAV is a promising tool for gene therapy [102].

### 3.3. Double-Stranded (ds) RNA Viruses

Most dsRNA viruses have viral genomes composed of 10–12 segments, and their capsids possess two layers but lack an envelope [103]. The process of generating daughter RNA involves copying the positive-strand RNA from the original negative strand using the template, followed by the new negative strand being copied from the positive-strand RNA. The Reoviridae family is the largest family of dsRNA viruses and includes a number of important human pathogens, such as rotaviruses and orthoreoviruses, which can cause severe gastroenteritis and respiratory infections, respectively. The unique genetic organization of these viruses and their ability to evade host immune responses present significant challenges for the development of effective antiviral therapies.

### 3.4. Positive-Sense (+) RNA Viruses

(+)ssRNA viruses can directly translate their genome into proteins using ribosomes, as their genome is similar in structure to mRNA [104]. Examples of (+)ssRNA viruses include DENV, ZIKV, norovirus, and the betacoronavirus SARS-CoV-2, which has caused the COVID-19 pandemic. SARS-CoV-2 has a spherical or elliptical shape with a diameter of 60–140 nm and is enveloped with a single-stranded positive-sense RNA genome. Its main components include the nucleocapsid protein (N), envelope protein (E), spike surface glycoprotein (S), and matrix protein (M). The S protein binds to cells and promotes viral entry into host cells [105]. The 3D structure of the SARS-CoV-2 S protein was successfully resolved by Wrapp [106], while the full-length structure of the cell surface receptor angiotensin-converting enzyme 2 (ACE2) was resolved by Yan [107].

### 3.5. Negative-Sense (−) RNA Viruses

Negative-sense single-stranded RNA (ssRNA) viruses [95] are a type of virus with a genome that consists of a negative-sense RNA. They require RNA-dependent RNA polymerase to synthesize positive-sense RNA within host cells, which is then used to generate negative-sense RNA. Because their genome is a negative-sense RNA, the replication of these viruses within host cells is dependent on RNA polymerase. The resulting RNA serves as mRNA for protein translation. The human influenza virus [108], a representative (−)ssRNA virus of the Orthomyxoviridae family, is spherical and typically consists of eight RNA segments of varying lengths. The viral ribonucleoprotein (vRNP) of the influenza virus, which is composed of RNA polymerase (PA, PB1, PB2) and the nucleoprotein (NP), plays a critical role in viral replication. The 3D structures of the NP protein and the heterodimeric structures of PA+PB1 and PB1+PB2 have been observed by Ye [109], Obayashi [110], and Sugiyama [111], respectively. In addition, Cusack et al. [112] proposed a detailed model of the entire transcription cycle of influenza polymerase in synthesizing mRNA based on the high-resolution cryo-electron microscopy structure of transcriptionally active bat influenza polymerase.

### 3.6. Reverse-Transcribing RNA Viruses

Retroviruses have a genome composed of two identical positive-sense ssRNA molecules, each containing long terminal repeat sequences that play a crucial role in the transcriptional regulation of viral DNA. The retroviral core contains reverse transcriptase and integrase. The human immunodeficiency virus (HIV) is a classic example of a retrovirus that is spherical or ellipsoidal with a diameter of 80–140 nm. The viral envelope is embedded with proteins gp120 and gp41, while inside is a spherical matrix formed by protein p17 and a semi-conical shell formed by protein p24 [113]. Studies have shown that both binding sites in gp41 may be associated with HIV infection of cells [114]. The co-infection of Hepatitis C virus (HCV) and HIV has been linked to faster development of hepatic fibrosis, higher rates of liver decompensation, and increased mortality rates in HIV-infected individuals compared to HCV mono-infection [115].

### 3.7. Double-Stranded DNA Retroviruses

After the initial six virus classifications were established in Baltimore, the Hepatitis B virus [116] (HBV) was discovered and subsequently classified as the seventh type of virus [95]. HBV [117] particles can exist in three different forms, which can be observed under electron microscopy as a large globular particle with a ~42 nm diameter, a small globular particle with a ~22 nm diameter, and a tube-shaped particle. Anastasiya et al. [118] discussed the potential clinical implications and fundamental importance of m6A RNA modification in HBV infection and pathogenesis.

## 4. Traditional Methods for the Detection of Viruses

### 4.1. Isolation and Culture Method

The isolation-and-culture method, which was first used to cultivate the cowpox virus, has undergone significant development and proven to be highly accurate in virus identification and testing, earning it the “gold standard” [119]. This method has made important contributions to the study of virus pathogenesis, vaccines, and drugs [120,121]. However, in many cases, medical teams require faster methods for pathogen detection to ensure optimal treatment outcomes. Therefore, this method may not apply to the rapid determination of most viruses.

### 4.2. Immunological Detection Methods

Immunological testing is a critical method for detecting pathogens by identifying specific antibodies produced by the human immune system. Common techniques in immunological testing include enzyme-linked immunosorbent assay (ELISA) and colloidal gold immunochromatography technology, both of which offer specificity and speed, making them valuable tools for rapid diagnosis and the surveillance of infectious diseases. ELISA [6,122,123] is widely used for detecting and quantifying antigens or antibodies in a variety of biological samples, while colloidal gold immunochromatography technology [124,125,126] is a simple and rapid diagnostic method that can detect specific proteins or antibodies in blood, urine, or saliva. Immunological detection has significantly contributed to the development of effective treatments and the diagnosis of infectious diseases.

While immunological techniques play a crucial role in virus detection, they do have inherent limitations [127], including the inability to detect low levels of viral antigens or antibodies in samples with low viral loads or during the early stage of infection. Additionally, the presence of non-target viruses or other substances can lead to false positive results, compromising the accuracy of the diagnosis. Moreover, prior knowledge about the specific antigens or antibodies associated with the virus being detected is typically necessary. Another limitation is the unavailability of information regarding the subtypes or mutations of the virus, which can hinder the accuracy of detection.

### 4.3. Nucleic Acid Amplification-Based Assays

Nucleic acid-amplification techniques are widely used in molecular biology to detect and amplify specific DNA or RNA sequences from biological samples. These techniques can be divided into two main categories: polymerase chain reaction (PCR) and isothermal amplification. PCR technology was developed in the 1980s and has become the broadest-used nucleic acid amplification technique because of its high sensitivity, specialization, and convenience [128]. Fluorescent PCR [129] and other PCR-based technologies have also been exploited for real-time monitoring of the amplification process, making it an important tool for the detection and diagnosis of infectious diseases. However, isothermal-amplification techniques do not require thermal cycling and can be completed using simple isothermal instruments. Examples include transcription-mediated amplification (TMA) [130], LAMP [3,131,132], etc. Isothermal-amplification techniques offer advantages such as faster and simpler protocols, making them particularly useful in resource-limited settings.

Although PCR and isothermal-amplification techniques have significantly contributed to virus detection, they still have certain limitations [133,134]. One primary challenge is the requirement for highly specialized experimental skills and sophisticated equipment, making these techniques less accessible. Moreover, as viruses undergo genetic mutations or deletions, there is a possibility that the primers used in PCR or isothermal amplification may not precisely match the target viral sequence. This mismatch can lead to a decline in amplification efficiency or even detection failure. Overcoming these limitations remains a significant challenge in advancing virus-detection technologies.

## 5. DNA Nanotechnology-Enabled Biosensor for Virus Detection

DNA nanotechnology represents a powerful technology for designing and building nucleic acid nanostructures in a “bottom-up” approach. For this nanotechnology, DNA molecules serve as structural materials, and their precise control at the nanoscale level enables the construction of “standardized” and “modular” tools, which facilitate the development of novel virus-detection platforms. By leveraging DNA nanotechnology, researchers can create customized and highly sensitive biosensors that can detect viruses with greater accuracy and efficiency. DNA nanomaterials obtained by using the self-assembly of DNA tiles have addressable characteristics and exhibit high sensitivity and affinity. These unique properties have laid the foundation for the development of DNA nanotechnology-based biosensors. Rapid progress in DNA nanotechnology has promoted the development of highly sensitive and precise virus-detection platforms.

DNA nanobiosensors [27,36] are sensing devices that utilize DNA nanotechnology and have the ability to convert the presence of target DNA into detectable electrical, optical, or acoustic signals. DNA nanobiosensors typically consist of two essential parts: a molecular-recognition element and a transducer. The molecular-recognition element is primarily responsible for sensing the amount of the target DNA in the sample, while the transducer converts the signal detected by the recognition element into observable and recordable signals. In DNA nanobiosensors, DNA molecules are used to construct programmable supramolecular nanostructures. These DNA nanostructures serve as templates to finely manipulate the spatial arrangement of sensing elements, leading to substantial enhancements in sensing performance and facilitating the development of sophisticated and novel biosensors [21,135,136]. Biosensors based on DNA templates adopt supramolecular DNA structures to serve as programmable anchor points. A notable example of these biosensors is DNA tetrahedron-based biosensors [20].

Recently, DNA nanobiosensors based on different types of technologies have been developed. Among them, optical and electrochemical DNA nanobiosensors have been mostly studied due to their high sensitivity, low cost, easy operation, short detection time, etc.

### 5.1. Optical DNA Nanobiosensors for Virus Detection

Optical DNA nanobiosensors can detect the presence of target DNA through optical signals, such as absorbance, reflectance, or fluorescence [137,138]. These biosensors typically use a recognition element, such as an ssDNA, that is immobilized on a surface and interacts with a complementary DNA strand to form a dsDNA. The interaction between the two strands results in a change in optical signal that can be detected and measured. According to the sensing mechanism, optical DNA biosensors can be generally categorized into surface-enhanced Raman scattering (SERS) biosensors, surface plasmon resonance (SPR) biosensors, and fluorescent biosensors (Table 1).

#### 5.1.1. SERS DNA Nanobiosensors

The gap between neighboring noble metal nanoparticles elicits an intensified electromagnetic field when they are close to each other, thereby resulting in a significant augmentation of the signal strength produced by Raman-active molecules [139,140]. SERS is intricately engineered to amplify Raman-scattering signals through the attachment of the analyte onto the surfaces of metal nanoparticles or nanostructures, thereby augmenting the Raman-scattering signal and facilitating its facile detection. SERS-based sensing techniques have garnered widespread application in the preceding decades owing to several merits: (i) Exceptional sensitivity; (ii) Capability for multiplex sensing; (iii) Suitability for point-of-care (POC) devices; and (iv) Ease of sample preparation [141,142,143,144]. The principal advantage of DNA nanobiosensors fabricated using SERS technology lies in their capacity to discern specific analytes even at exceedingly low concentrations [145]. The primary challenge impeding the progress of SERS-based DNA nanobiosensors is attributed to the requirement for close contact between the analyte and the surface, as prolonged usage may lead to a partial loosening of this contact, consequently diminishing the signal intensity [146]. To meet the escalating need for precise and expeditious virus detection, SERS-sensing technologies have demonstrated great potential for multiple immunoassays [147].

**Figure 4:**
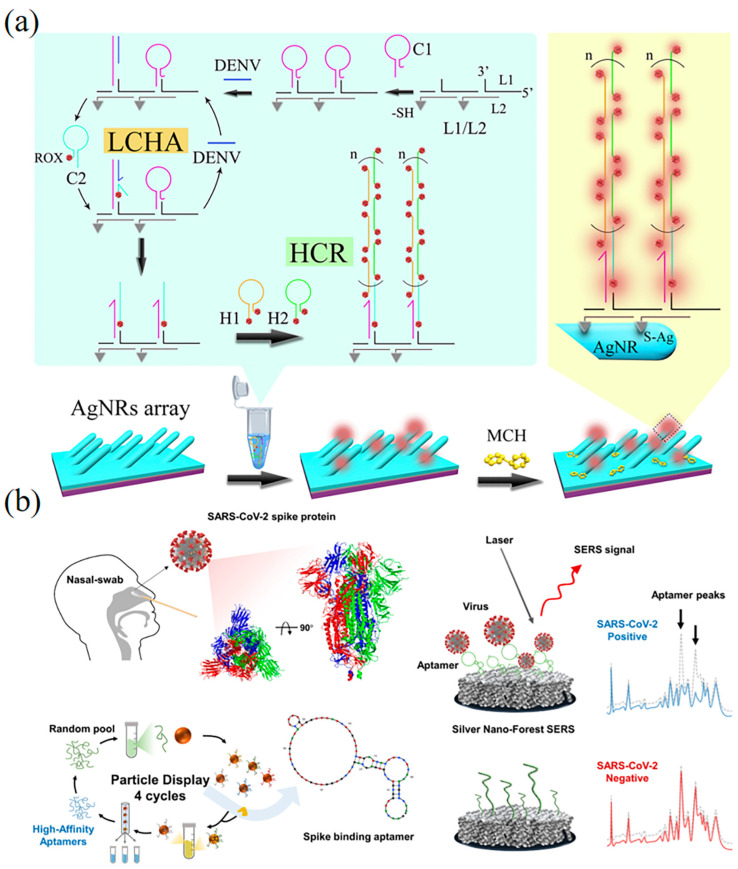
SERS DNA nanobiosensors for virus detection. (**a**) Schematic diagram of SERS biosensor for DENV gene by cascade enzyme-free signal amplification strategy of local catalytic hairpin assembly (LCHA) and hybridization chain reaction (HCR) [148]. Copyright 2020, Elsevier. (**b**) SERS-based design strategy for the SARS-CoV-2 label-free sensitive-sensor platform [150]. Copyright 2023, Elsevier.

Song et al. designed a locally catalyzed hairpin assembly (LCHA) and hybridization chain reaction (HCR) SERS sensor from DNA tiles (Figure 4a) [148]. The DNA sequence of the dengue virus (DENV) was identified by an LCHA system consisting of L1 and L2 strands and Hairpin C1. When the ROX dye-labeled Hairpin C2 was introduced, it can self-assemble with the L1, L2, and C1 strands to form LCHA, and the DNA sequence of DENV can be continuously recycled. Additionally, ROX-labeled hairpins (H1 and H2) were added to the SERS-AgNRS array, initiating HCR and enhancing the signal of ROX. The SERS intensity of this biosensor combining of LCHA and HCR is about 2.8 times that of the single LCHA strategy, and even more than 4.5 times that of the conventional CHA strategy. Meanwhile, the signal-to-noise ratio (S/N, I_DENV_/I_blank_) of LCHA–HCR exhibits 5.4 times that of individual CHA. The limit of detection (LOD) for DENV is as low as 0.49 fM, which is much higher than the previous SERS sensor [149].

Park et al. have developed a sensitive label-free SERS aptamer sensor for detecting SARS-CoV-2 variants of interest (Figure 4b) [150]. Using a high-throughput screening method, they identified two DNA aptamers that could bind to the SARS-CoV-2 spike protein with an affinity of 1.47 ± 0.30 nM and 1.81 ± 0.39 nM. Leveraging these aptamers in conjunction with silver nanoforests, they designed an ultrasensitive SERS platform, achieving a detection limit of 10^−18^ M for recombinant trimeric spike proteins. Moreover, they demonstrated a label-free aptamer sensor that does not require Raman labels, utilizing the intrinsic signals of the aptamer. Notably, the label-free SERS sensor exhibited outstanding accuracy in clinical samples encompassing wild-type, Delta, and Omicron variants.

#### 5.1.2. Surface Plasmon Resonance-Based DNA Nanobiosensors

The operating principle of surface plasmon resonance (SPR)-based DNA nanobiosensors relies on detecting alterations in the refractive index occurring at the interface between the dielectric layer and the metal layer [151,152]. The SPR response is intricately connected to the characteristics of the metal layer, with gold being the most favorable choice [153]. Classical SPR sensors primarily comprise a stationary recognition element, an optical prism, and an analyte [154]. In an SPR-based DNA nanobiosensor, the engineered DNA nanostructures are initially immobilized onto the surface of a metal film. Following surface functionalization, a sample solution containing analytes is then introduced onto the surface. As incident light strikes the medium at varying angles, photons are absorbed by the plasma wave at the critical angle, which is influenced by the refractive index of the medium. When the nucleic acid of a virus sample interacts with DNA nanomaterials, the refractive index of the medium in the vicinity of the metal film’s surface undergoes alteration, leading to a resonant angular shift of the plasma wave, thus facilitating virus detection [155]. The SPR-based DNA nanosensors have demonstrated remarkable promise in enabling rapid POC virus detection, owing to their sensitive and label-free detection mechanism [156]. SPR-based DNA nanobiosensors exhibit excellent accuracy in virus detection, and they also offer the advantages of label-free monitoring, rapidity, and sensitivity. However, to harness the full potential of SPR technology for early diagnosis of viruses, further enhancements in specificity and sensitivity are still required [157].

**Figure 5:**
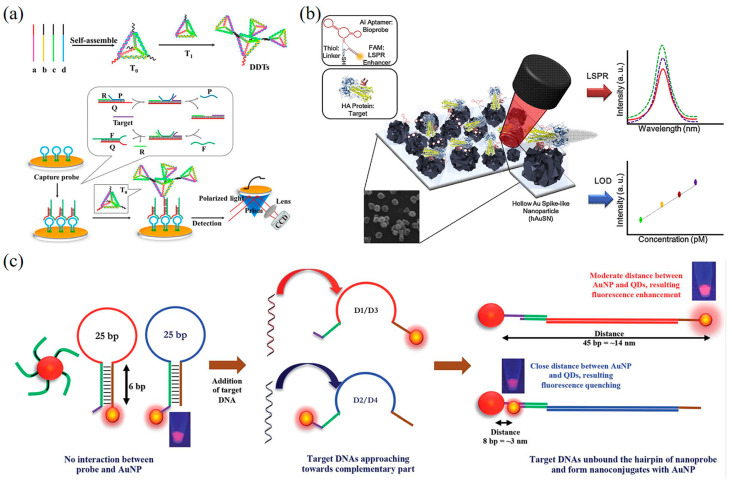
SPR DNA nanobiosensors for virus detection. (**a**) Schematic diagram of SPR biosensors based on ESDRs and DDTs nanostructures for the detection of HIV-related DNA [158]. Copyright 2017, Elsevier. (**b**) Diagram of the AIV detection biosensor fabricated based on the LSPR method [159]. Copyright 2019, Elsevier. (**c**) Schematic of the mechanism of DENV detection by AuNPs and hairpin ssDNA–CdSeTeS QDs [160].

Wei et al. developed a SPR DNA biosensor through entropy-driven strand displacement reaction (ESDR) and dsDNA tetrahedron (DDT) for monitoring HIV-related DNA (Figure 5a) [158]. Target DNA can specifically trigger the enzymatic-signal amplification circuits to form numerous dsDNA, which bind to the hairpin capture probes and interact with DDT nanostructures. The SPR DNA biosensor can detect target DNA in a linear range from 150 nM to 1 pM with an LOD of 48 fM. Lee et al. developed a label-free biosensor for detecting avian influenza (AIV H5N1) using localized surface plasmon resonance (LSPR) technology (Figure 5b) [159]. The biosensor consists of a multifunctional DNA three-way junction (3WJ) located on a hollow gold spike-shaped nanomaterial, which demonstrated the ability to detect AIV and other viruses. SPR biosensors have the advantage of label-free detection, simplified molecular hybridization, and short time. In comparison, SPR biosensors do not require the use of labels, such as fluorescence or radioisotopes, to detect the target molecule with less influence on biological molecules, making it simpler due to the sample preparation and short time for detection.

Chowdhury et al. developed a DNA nanobiosensor for the rapid and quantitative detection of all four serotypes of dengue virus by exploiting the distance-based localized surface plasmon resonance (LSPR) effect between cadmium telluride selenide fluorescent quantum dots (CdSeTeS QDs) and gold nanoparticles (AuNPs) (Figure 5c) [160]. In this study, they designed four nanoprobes that were covalently linked to serotype-specific hairpin ssDNA primers at different positions on the CdSeTeS QDs. The hairpin structure featured a self-complementary anchoring region composed of six polyguanines (poly-G) and polycytosines (poly-C) bound to CdSeTeS QDs. Additionally, thiolated poly-C functionalized AuNPs were also synthesized. To detect DENV serotypes, both synthetic ssDNA and real RNA samples were employed in the study. The target ssDNA/RNA sequences of DENV were skillfully hybridized to the complementary ssDNA loop sequence of the hairpins. This manipulation led to the opening of the complementary ssDNA loop sequence, resulting in the formation of a linear ssDNA probe strand conjugated to QDs. Subsequently, the target DNA/RNA sequences were precisely bound to the nanoprobe via complementary binding. The distance effect based on LSPR enabled the successful rapid and quantitative detection of DENV serotypes. The method demonstrated remarkable sensitivity with a LOD of 24.6 fM for DENV1, 11.4 fM for DENV2, 39.8 fM for DENV3, and 39.7 fM for DENV4, respectively. The DNA nanobiosensor holds great promise for practical applications in the detection of dengue virus serotypes with exceptional accuracy and efficiency.

#### 5.1.3. Fluorescence-Based DNA Nanobiosensors

The fluorescence-based DNA nanobiosensor relies on detecting changes in fluorescence signals upon the binding of a fluorescent dye-labeled DNA probe to the target molecule [161,162,163]. The fluorescent dye labeled on the DNA probe typically remains unexcited when not bound to the target molecule. Upon the specific pairing of the DNA probe with the target molecule, the spatial location of the fluorescent dye changes, leading to the change of fluorescence signals and facilitating the detection of the target molecule. The fluorescence-based DNA nanobiosensor has high spatial and temporal resolution, high sensitivity, and short response time, making it suitable for multiplexed assays [164]. These advantages render the fluorescence-based DNA nanobiosensor a desirable and effective sensing tool for virus detection and other biomolecule analysis [165,166,167]. Nevertheless, fluorescence-based DNA nanobiosensors may encounter limitations such as fluorophore scintillation or photobleaching, which can render them unsuitable for certain applications. Additionally, the issue of non-specific binding of fluorescent markers to other environmental components continues to challenge their implementation [168,169,170].

**Figure 6:**
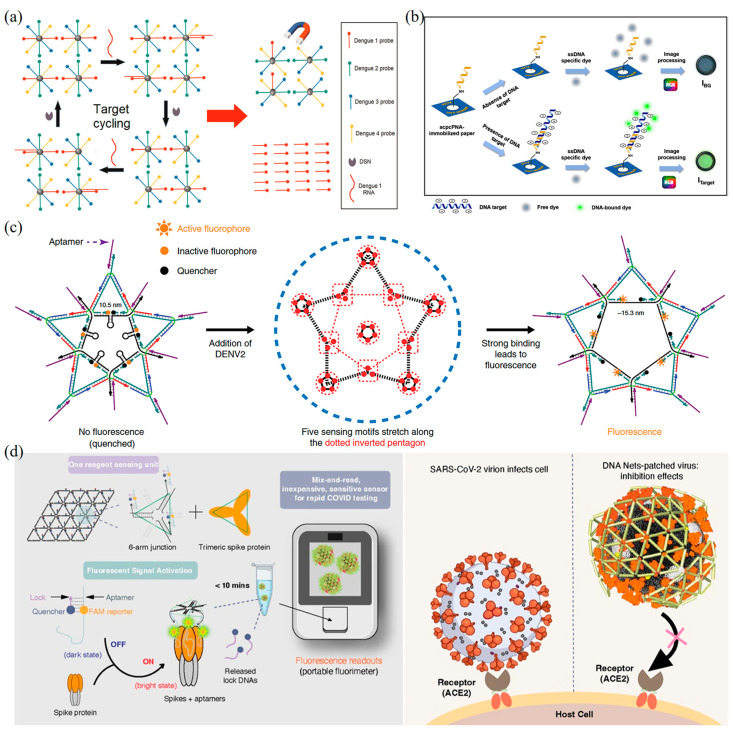
Fluorescence-based DNA nanobiosensors for virus detection. (**a**) Schematic representation of the working principle of the fluorescence DENV assay based on QD-CPs [171]. Copyright 2015, Elsevier. (**b**) The working principle of the fluorescence-based detection of HCV using the acpcPNA-immobilized PAD and ssDNA-specific fluorescence dye [173]. Copyright 2021, Elsevier. (**c**) Dimensional analysis and design concept of the DNA tile structure corresponding to the ED3 cluster on the DENV surface [175]. Copyright 2019, Springer Nature. (**d**) Schematic design concept and detection of DNA mesh structures binding to SARS-CoV-2 [31]. Copyright 2019, Springer Nature.

Shen et al. developed a fluorescent DNA biosensor to detect four types of DENV using a quantum dot-capped DNA capture probe (QD-CPs) (Figure 6a) [171]. In this biosensor, DNA capture probes bind to the surface of quantum dots and magnetic beads. During detection, DENV ssRNA forms heterologous double strands with the DNA capture strand. Subsequently, double-strand-specific nuclease (DSN) cleaves the DNA capture probe and releases quantum dots from magnetic beads. The DENV ssRNA can continue to hybridize with the remaining DNA capture probe. The ultrasensitive detection of DENV is achieved through the fluorescence recovery of quantum dots through the DSN cleavage process. The method achieves an LOD of 0.5 fM, which is four orders of magnitude higher than previous studies [172].

Teengam et al. developed the paper-based fluorescent DNA biosensor by using acpcDNA for the selective detection of the Hepatitis C virus (HCV) (Figure 6b) [173]. The biosensor demonstrated a linear correlation between fluorescence changes and the amount of HCV DNA, with an LOD of 5 pM. The biosensor is highly selective for complementary oligonucleotides rather than complementary targets and has been successfully applied to detect HCV complementary DNA (cDNA) obtained from clinical samples. Jiao et al. presented a DNA nanoscaffold hybridization chain reaction (DNHCR)-based nucleic acid assay for rapid detection of SARS-CoV-2 [174]. Upon the presence of the target SARS-CoV-2 RNA, an intricate cascade reaction is initiated within the DNA nanoscaffold, leading to H1 separation and consequent fluorescence recovery. Notably, this process enables localized acceleration of the DNA probe, resulting in the immediate and highly amplified fluorescence-signal generation throughout the entire nanoscaffold in response to a single target RNA molecule. This innovative DNHCR assay demonstrates high versatility, as it can be effectively executed in serum and saliva samples within a remarkably short detection time (within 10 min) and a suitable temperature range of 15–35 °C.

Chao et al. designed fluorescent DNA biosensors that match the surface antigen of DENV and combined them with nucleic acid aptamers to achieve fluorescence detection and activity inhibition of DENV (Figure 6c) [175]. They demonstrated that 2D spatial-pattern recognition is essential for the binding of DNA nanostructures to targets, providing important insights for improving the binding between known ligands and DNA nanostructures. These fluorescence DNA biosensors have the potential to be used in immediate healthcare applications due to their sensitivity, specificity, and ease of use. Ochmann et al. effectively achieved enhanced fluorescence signals for target nucleic acids by employing DNA folding-based optical antennas in conjunction with metal nanoparticles, leveraging plasma effects, and generating specific signals [176]. As exemplified in the detection of the Zika virus, this method successfully demonstrates the ability to detect Zika-specific artificial DNA and RNA in buffer solutions and heat-inactivated human serum, displaying sensitivity to small nucleotide variations that enable discrimination between related pathogens. Moreover, the modular nature of DNA nanotechnology enabled the parallel detection of multiple fluorescent markers. The signal-enhancing strategy presented in this study holds promise for simplifying signal detection approaches in single-molecule base-point diagnostics. Chowdhury et al. constructed a fluorescent DNA biosensor for quantifying four DENV [177]. In this study, AuNP-graphene quantum dots nanocomposites (GQD-AuNP) were linked with four dye-labeled DNA probes. The results demonstrated that GQD-AuNP can effectively detect four serotypes of DENV in the concentration ranging from 10^−14^ to 10^−6^ M with an LOD of 9.4 fM. The sensor also showed satisfactory performance in clinical applications for DENV detection.

Chauhan et al. have successfully presented a novel mesh DNA nanostructure, named “DNA Net”, which exhibits the capability to selectively recognize and capture intact SARS-CoV-2 viruses (Figure 6d) [31]. This DNA Net is ingeniously designed to incorporate aptamers that specifically target the viral surface spike glycoproteins. Upon binding to the viruses, the DNA Net aptamers elicit the release of a fluorescent signal, facilitating swift and sensitive COVID-19 detection assays. Furthermore, the authors have demonstrated that the DNA Net aptamers can accomplish virus detection in various modes, notably through the fluorescence signal, enabling rapid and sensitive COVID-19 detection assays. The LOD for the 4 × 4 DNA sensor is comparable to the clinical SARS-CoV-2 viral load (ranging from 1 × 10^4^ to 1 × 10^10^ copies/mL in the upper respiratory tract). Notably, this sensor directly recognizes intact virus in a sample, obviating the need for nucleic acid material extraction, RNA purification, enzyme amplification, thermal cycling, and complex equipment calibration. As a result, a low-cost, rapid, and sensitive assay can be conducted isothermally at room temperature, providing easily interpretable results. Moreover, by directly detecting intact SARS-CoV-2 virus particles, this method can address inquiries concerning a patient’s infectious status and potential release from isolation, adding to its clinical relevance.

Gogianu et al. presented a proof-of-concept for an advanced microarray platform designed to enhance DNA detection [178]. In this study, three-dimensional microarray chips were fabricated using metal-assisted chemical etching on silicon nanowire substrates, subsequently coated with SU-8 polymer and further modified with carbon quantum dots (CDs) in either poly (dimethyldiaminomethane acrylate) (PDDA) or polyethyleneimine (PEI) solutions. High-quality-labeled HPV 16-targeted ssDNA was hybridized to the SiNWs/SU-8/CDs platform. Notably, the hybridized DNA exhibited a strong fluorescence signal when attached to the SiNWs/SU-8, with the further amplified signal by the inclusion of CDs. Optimal signal intensity and coefficient of variation were achieved through co-immobilization of the HPV 16 probe by functionalizing CDs with PDDA.

**Table 1:** DNA nanotechnology-enabled biosensors for optical detection of viruses.

Detection Technique	Target Pathogen	DNA Nanoprobe	LOD	Reference
SERS nanobiosensors	DENV	DNA HCR probe	0.49 fM	[148]
	SARS-CoV-2	DNA aptamer	1 pM	[150]
SPR nanobiosensors	HIV	Double-layer DNA tetrahedrons	48 fM	[158]
	AIV H5N1	DNA 3 way-Junction	-	[159]
	DENV	DNA hairpin	24.6 fM (DENV-1) 11.4 fM (DENV-2) 39.8 fM (DENV-3) 39.7 fM (DENV-4)	[160]
Fluorescent nanobiosensors	SARS-CoV-2	Net-Shaped DNA Nanostructures	1 × 10^8^ viral genome copies/mL	[31]
	DENV	Quantum dot-capped DNA capture probes	0.50 fM	[171]
	HVC	acpcPNA-DNA double helix	5 pM	[173]
	DENV	DNA star	1 × 10^2^ p.f.u.mL^−1^ (serum) 1 × 10^3^ p.f.u.mL^−1^ (plasma)	[175]
	SARS-CoV-2	DNA nanoscaffold	0.96 pM	[174]
	ZIKV	DNA nanoantenna	-	[176]
	DENV	DNA double helix	9.4 fM	[177]
	HPV-16	DNA-based microarray biochip	-	[178]

### 5.2. Electrochemical DNA Nanobiosensors for Virus Detection

In electrochemical DNA biosensors, ssDNA probes with recognition ability are immobilized onto an electrode and can hybridize target ssDNA, resulting in the formation of a dsDNA. This process occurs on the surface of the electrode and influences the electrical signals, such as current, potential, and impedance, which can be used to quantize the amount of target DNA. By immobilizing DNA probes on a substrate surface, electrochemical biosensors can recognize complementary target sequences and convert sequence-recognition hybridization signals into electrical signals through a signal transduction device. The electrical signals can then be classified as amperometric and resistive sensors based on the electrical quantity they convert (Table 2). These real-time monitoring and on-site rapid-detection features make electrochemical DNA biosensors a promising POC testing technology for virus detection [179]. They offer high sensitivity, simplicity, low cost, and ease of miniaturization, making them an attractive option for the development of portable diagnostic devices.

#### 5.2.1. Voltammetry-Based DNA Nanobiosensors

Voltammetry is a technique that utilizes voltage ramps to measure the change in current, generating distinctive oxidation and reduction peaks for each analyte. In recent years, researchers have focused on cyclic voltammetry (CV), differential pulse voltammetry (DPV) [180,181], and square-wave voltammetry (SWV) [182] to detect different viruses. For DPV, a differential potential pulse is administered with a constant amplitude while continuously increasing the scanning potential. The current of each pulse is divided into two phases: before the potential application and at the end of the pulse. The difference between these two currents is then plotted as a function of the potential [183]. SWV is a differential voltammetry technique characterized by large-amplitude pulses, where a constant-amplitude pulse is applied while the scanning potential continuously increases. The potential-time plot in SWV is derived by measuring and subtracting the current at the end of the pulse from the current at the beginning of the pulse, and the resultant difference in flow is plotted against the increase in scanning potential [184,185]. The advantage of SWV over DPV lies in its high speed.

**Figure 7:**
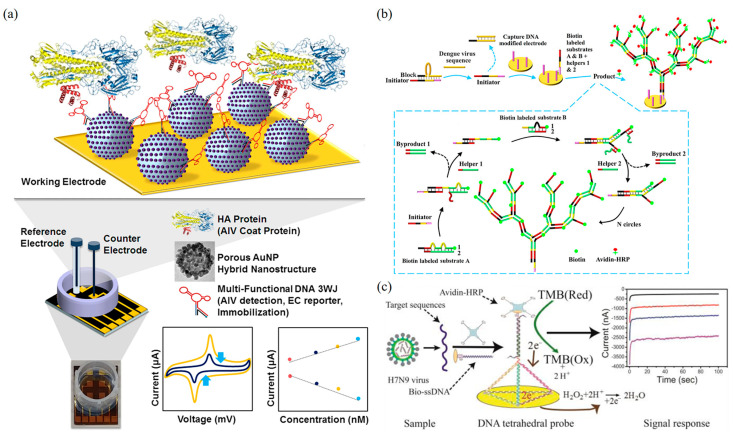
Electrochemical DNA nanobiosensors for virus detection. (**a**) Schematic image of the fabricated AIV detection biosensor [186]. Copyright 2019, Elsevier. (**b**) Schematic illustration of triplet nanostructure-mediated dendritic HCR for electrochemical detection of DENV [187]. Copyright 2021, Elsevier. (**c**) Schematic diagram of an electrochemical biosensor based on DNA tetrahedral nanostructures for the detection of H7N9 virus [188]. Copyright 2015, American Chemical Society.

Lee et al. developed a DNA-based biosensor using label-free porous gold nanoparticles (pAuNPs), which is capable of detecting the H5N1 virus (Figure 7a) [186]. The electrodes were coated with pAuNPs to increase the surface roughness and electron transfer effect. DNA three-way junctions (3WJs) consist of three fragments, including HA protein detection (recognition fragment), electrochemical signal generation (DNAzyme), and immobilization parts (thiol group), which were introduced on the Au electrode. The LOD of this biosensor is 9.43 pM in HEPES buffer and 1 pM in diluted chicken serum. Ju et al. developed a highly sensitive and versatile electrochemical biosensing strategy for the detection of DENV nucleic acids (Figure 7b) [187], which involves immobilizing trapped DNA on the electrode through a dendritic hybridization chain reaction (HCR). The target DENV DNA sequence first identifies the dsDNA in the block state and releases the initiator strand. Then, the gold electrode modified with capture DNA captures the initiator strand, resulting in a triplet-state nanostructure. Finally, a tree-like HCR is triggered after the introduction of auxiliary Strand 2 to form the branching DNA nanostructure, creating an amplified current signal. The linear detection range of this method for DENV is 1.6 to 1000 pM, with a LOD of 188 fM. Importantly, the method can distinguish single-base mutations.

Song et al. developed an electrochemical biosensor based on DNA tetrahedral nanostructures for the detection of the H7N9 virus by recognizing fragments of the hemagglutinin gene sequence (Figure 7c) [188]. In this biosensor, a DNA tetrahedral probe was immobilized on the surface of a gold electrode, and the hybridization to the target ssDNA was achieved by self-assembly of three thiol-modified nucleotide sequences with longer nucleotide sequences containing complementary DNA. The captured target DNA strands hybridize to a biotinylated ssDNA probe followed by the addition of the affinity hormone horseradish peroxidase to generate an amperometric signal by reacting with a 3, 3′, 5, 5′-tetramethylaniline substrate. The electrochemical biosensor specifically recognizes the target DNA of the H7N9 virus, which can be differentiated from other kinds of influenza viruses (e.g., H1N1 and H3N2) as well as from single-base mismatched oligonucleotides. The LOD of this biosensor for H7N9 can reach as low as 100 fM. This study also showed that electrochemical biosensors based on DNA tetrahedral probes can effectively detect the target DNA in clinical samples.

Mahmoodi et al. described a DNA-based selective and sensitive electrochemical biosensor for the early detection of HPV-18 [189]. By electrodeposition of reduced graphene oxide (rGO) and multi-walled carbon nanotubes (MWCNTs) on printed carbon electrodes, followed by dropwise addition of gold nanoparticles (AuNPs) on the modified electrodes and immobilization of ssDNA probes on the modified electrode. DPV was used performed to detect HPV-18 by monitoring the change in the oxidation signal of anthraquinone sulfonate (AQMS) before and after the hybridization of the probe to the target DNA. The experimental results showed that the biosensor was linear over the concentration range from 0.01 fM to 0.01 nM, with the lowest limit of detection of 0.05 fM. The DNA nanobiosensor also showed good sensing performance for clinical samples.

#### 5.2.2. Impedance-Based DNA Nanobiosensors

In contrast to the direct current (DC) measurement used in voltammetry, impedance-based sensors utilize the changes in impedance to detect the presence of target molecules [190,191]. In impedance-based DNA nanobiosensors, biomolecules interact with DNA probes on the sensor surface to form DNA duplexes. When an alternating electric field is applied, the process of duplex formation and decomposition impacts the current through the sensor, leading to a change in impedance. By measuring this change, the presence of the target molecule and its concentration can be determined [192]. The impedance-based biosensor usually exhibits a low LOD in the picomolar (pM) range and demonstrates good stability, making it an optimal choice for virus detection [193].

**Figure 8:**
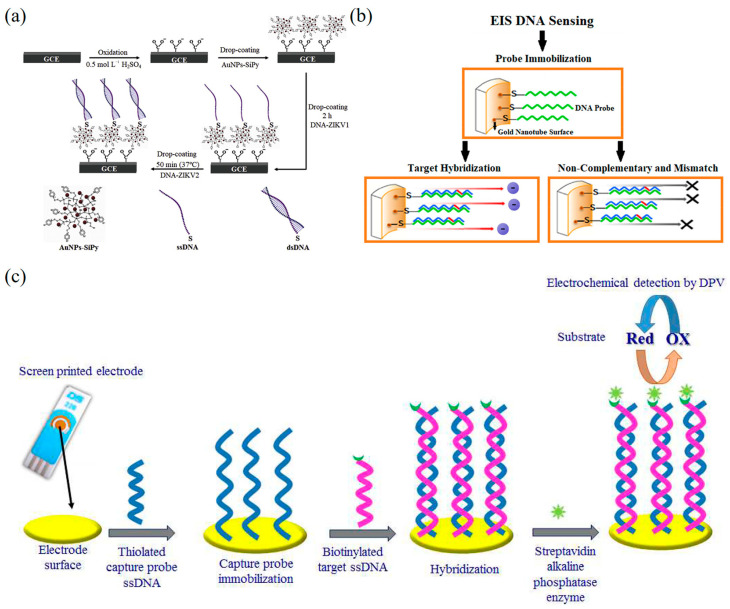
Impedance-based DNA nanobiosensors for virus detection. (**a**) Schematic representation of the construction and DNA hybridization stages of the oxidized-GCE-[AuNP-SiPy]/ZIKV1 biosensor [195]. Copyright 2019, Elsevier. (**b**) Schematic representation of different steps for the fabrication of electrochemical DNA biosensors [196]. Copyright 2018, Elsevier. (**c**) AuNT PC surface probe immobilization and hybridization of HPV-DNA target sequences with AuNT surface modifications and schematic diagrams [197]. Copyright 2019, Elsevier.

Shariati et al. developed a label-free electrochemical impedance DNA biosensor for HBV detection using gold nanocrystals (AuNCs) [194]. The biosensor was constructed by immobilization of the HBV DNA probe (ssDNA) on the surface of AuNCs. EIS study showed that the LOD 0.1 fM can be achieved for the HBV DNA. Moreover, it is possible to distinguish between complementary and non-complementary DNA targets, including one, two, and three mismatch targets. The biosensor ability also showed the ability to detect HBV in serum samples. Wang et al. developed an electrochemical impedance spectroscopy (EIS)-based DNA sensor for the detection of HBV and human papillomavirus (HPV) [192]. The sensor utilizes ssDNA probes that self-assemble on AuNP-coated single-walled carbon nanotubes (SWCNT/Au). The LOD of this biosensor is 0.1 pM for HBV and 1 aM for HPV, respectively. Additionally, this biosensor exhibits excellent stability, ensuring reliable and consistent results over time. Steinmetz et al. developed a DNA biosensor for Zika virus (ZIKV) in human serum samples by utilizing silsesquioxane-functionalized gold nanoparticles (AuNPs-SiPy) to modify an oxidized glassy carbon electrode (ox-GCE) (Figure 8a) [195]. The biosensor detects ZIKV by measuring changes in the charge transfer resistance (ΔR_ct_) and the electrode surface roughness (Rq). The biosensor reached an LOD as low as 0.82 pM and can detect the target in a linear range of 1.0 × 10^−12^–1.0 × 10^−6^ M.

Ilkhani et al. developed a novel electrochemical DNA nanobiosensor for the detection of Ebola virus DNA, utilizing an enzymatic amplification assay to enhance the sensitivity and selectivity (Figure 8b) [196]. To construct the nanobiosensor, a sulfhydrylated DNA capture probe is immobilized onto a printed electrode and hybridized with a biotinylated target strand DNA. To facilitate electrochemical detection, the biotinylated hybridization product was labeled with streptavidin-alkaline phosphatase conjugate on the surface of the working electrode, allowing subsequent enzymatic product detection through DPV. To optimize the entire experimental procedure, electrochemical impedance spectroscopy (EIS) was employed, successfully achieving favorable conditions for biosensor preparation. The electrochemical DNA nanobiosensor with an LOD of 4.7 nM was achieved for Ebola virus DNA, demonstrating its potential as a sensitive detection tool.

Zucolotto et al. demonstrated an electrochemical DNA nanobiosensor that relies on an impedance method for label-free detection of the Zika virus [198]. The biosensor employs disposable electrodes fabricated through thermal evaporation on a polyethylene terephthalate substrate, coated with a nanoscale gold layer. A three-contact electrode configuration is utilized to identify DNA sequences in a small sample volume, eliminating the need for labeling in the detection of Zika virus sequences. The nanobiosensor exhibited a response time of 1.5 h and achieved an LOD of 25.0 ± 1.7 nM through impedance measurements. The nanobiosensor exhibited selectivity for Zika virus to synthetic DNA.

Shariati et al. demonstrated a label-free DNA nanobiosensor for HPV detection, constructed by employing gold nanotubes (AuNTs) (Figure 8c) [197]. AuNT-modified nanoporous polycarbonate (AuNT-PC) templates were prepared through an electrodeposition method, while ssDNA probes were covalently immobilized onto the AuNT-PC electrodes. Hybridization between the target DNA sequence and the ssDNA probe was precisely monitored using the EIS technique. The nanobiosensor exhibited remarkable selectivity among complementary, mismatched, and non-complementary DNA sequences. The nanobiosensor demonstrated outstanding performance for the detection of HPV DNA with an LOD of 1 fM and a linear response range from 0.01 pM to 1 μM.

**Table 2:** DNA nanotechnology-enabled biosensors for electrochemical detection of viruses.

Detection Method	Target Pathogen	DNA Nanoprobe	LOD	Reference
Voltammetry biosensors	H5N1	DNA 3 way-Junction	1 pM	[186]
	DENV	DNA double helix	43 nM	[199]
	H1N1	DNA aptamer	3.7 PFU/mL	[200]
	DENV	DNA HCR	188 fM	[187]
	H7N9	DNA tetrahedral	100 fM	[188]
	H5N1	DNA double helix	1.39 pM	[182]
	HPV-18	Cys-AuNPs-DNA probe	0.05 fM	[189]
Impedance biosensors	HBV	ssDNA/AuNCs	0.1 fM	[194]
	HBV	SWCNTs/Au/ssDNA	0.1 pM	[192]
	ZIKV	DNA double helix	0.82 pM	[195]
	Ebola	DNA capture probe	4.7 μM	[196]
	ZIKV	DNA HCR	25 nM	[198]
	HPV	DNA aptamer	1 fM	[197]

## 6. Summary and Outlook

This review provides a brief review of DNA nanotechnology, virus classification, and the optical and electrochemical DNA biosensors for virus detection. The advantages of DNA nanotechnology in the construction of nanobiosensors have been highlighted, including their designability, addressability, and bioaffinity, which make them valuable tools for virus detection. Furthermore, biosensors based on DNA nanotechnology for virus detection are discussed. The working principles of typical DNA-enabled biosensors are described. The recent progress made in virus detection by optical DNA biosensors and electrochemical DNA biosensors is discussed in detail.

DNA nanobiosensors for the virus have the following advantages over conventional methods. (i) High specificity: DNA nanobiosensors can recognize target molecules by sequence-specific base pairing and thus have a high degree of selectivity. This makes it possible to accurately detect target substances and avoid false positives and interference. (ii) High sensitivity: DNA nanobiosensors are capable of single-molecule-level detection with high sensitivity. With appropriate signal amplification and enhancement strategies, target viruses with low concentrations can be detected. (iii) Programmability and tunability: DNA is a programmable biomolecule that can be designed to synthesize specific DNA sequences to achieve diverse sensor functions. By adjusting the combination, length, and structure of DNA sequences, the performance of the sensor can be customized and optimized. (iv) Biocompatibility: DNA nanobiosensors are biocompatible in living organisms. DNA is one of the naturally occurring molecules in living organisms, and the use of DNA as a sensor material can reduce toxicity and immune response to living organisms. (v) Fast response and real-time monitoring: DNA nanobiosensors have the characteristics of fast response and can complete the detection of target substances in a short time. Meanwhile, due to its highly sensitive characteristics, it can achieve real-time monitoring and rapid feedback of changes in the target substance.

Although biosensors based on DNA nanotechnology have demonstrated great potential for virus detection, some challenges still exit in this newly emerging research area. First, a fundamental understanding of the relationship between DNA nanostructure geometry and sensor performance needs further study. Due to the flexibility of design, DNA nanostructures can be accurately prepared with tunable shapes and sizes, which provides a great opportunity for the mechanistic study of signal transduction in the sensors. Second, the complexity of DNA nanostructures served as sensing elements should be further simplified. Third, the negative charge of DNA nanostructures may hinder nucleic acid hybridization, affecting the performance of DNA biosensors [201]. Fourth, the current study is mainly performed at the lab level, and there are still unresolved issues before clinical translation, such as the stability of DNA nanostructure in body fluids and the mass production of DNA nanostructure-based biosensors.

Despite these challenges, researchers have made great efforts to address these issues [175]. For example, Praetorius [202] proposed a method using phages to generate DNA precursors for large-scale and cost-effective DNA origami preparation while maintaining customizability. DNA nanostructures also offer opportunities for targeted therapies [72] and transport across cell membranes [203], which could enable real-time monitoring and suppression of viruses within the human body. Combining DNA biosensors with deep learning and neural network technology can develop more intelligent biosensors with improved accuracy and sensitivity. As DNA nanotechnology continues to advance, the existing barriers to clinical applications will finally be overcome.

In conclusion, DNA nanobiosensors have demonstrated great promise for virus detection and have achieved notable advancements. Nevertheless, further endeavors are still needed to address many challenges before the clinical translation of DNA nanobiosensors. For instance, improving the stability and longevity of DNA nanobiosensors is crucial to ensure their reliability. The anti-interference of DNA nanobiosensors in complicated samples and environments should be further improved, which is important for practical applications. Additionally, issues of high technological thresholds and relatively high costs involved in the design and synthesis process of DNA nanostructures should be solved. By persistently pursuing in-depth research and technological innovation, DNA nanobiosensors are poised to unlock new possibilities in virus detection and other biomedical fields. Their potential to contribute to personalized and precision medicine is highly prospected. The ongoing pursuit of advancements in this field holds the promise of revolutionizing diagnostic capabilities, enabling more accurate and tailored approaches to healthcare, and ultimately, positively impacting human health.

## Data Availability

Not applicable.

## References

[1] Ke Z., Oton J., Qu K., Cortese M., Zila V., McKeane L., Nakane T., Zivanov J., Neufeldt C.J., Cerikan B. (2020). Structures and Distributions of SARS-CoV-2 Spike Proteins on Intact Virions. Nature.

[2] Yao H., Song Y., Chen Y., Wu N., Xu J., Sun C., Zhang J., Weng T., Zhang Z., Wu Z. (2020). Molecular Architecture of the SARS-CoV-2 Virus. Cell.

[3] Notomi T. (2000). Loop-Mediated Isothermal Amplification of DNA. Nucleic Acids Res..

[4] Smyrlaki I., Ekman M., Lentini A., de Sousa N.R., Papanicolaou N., Vondracek M., Aarum J., Safari H., Muradrasoli S., Rothfuchs A.G. (2020). Massive and Rapid COVID-19 Testing Is Feasible by Extraction-Free SARS-CoV-2 RT-PCR. Nat. Commun..

[5] Yelin I., Aharony N., Tamar E.S., Argoetti A., Messer E., Berenbaum D., Shafran E., Kuzli A., Gandali N., Shkedi O. (2020). Evaluation of COVID-19 RT-QPCR Test in Multi Sample Pools. Clin. Infect. Dis..

[6] Deshpande K., Pt U., Kaduskar O., Vijay N., Rakhe A., Vidhate S., Khutwad K., Deshpande G.R., Tilekar B., Saka S. (2021). Performance Assessment of Seven SARS-CoV-2 IgG Enzyme-linked Immunosorbent Assays. J. Med. Virol..

[7] Dewald F., Suárez I., Johnen R., Grossbach J., Moran-Tovar R., Steger G., Joachim A., Rubio G.H., Fries M., Behr F. (2022). Effective High-Throughput RT-QPCR Screening for SARS-CoV-2 Infections in Children. Nat. Commun..

[8] Chen Y., Chan K.-H., Kang Y., Chen H., Luk H.K.H., Poon R.W.S., Chan J.F.W., Yuen K.-Y., Xia N., Lau S.K.P. (2015). A Sensitive and Specific Antigen Detection Assay for Middle East Respiratory Syndrome Coronavirus. Emerg. Microbes Infect..

[9] Amanat F., Stadlbauer D., Strohmeier S., Nguyen T.H.O., Chromikova V., McMahon M., Jiang K., Arunkumar G.A., Jurczyszak D., Polanco J. (2020). A Serological Assay to Detect SARS-CoV-2 Seroconversion in Humans. Nat. Med..

[10] Ye Q., Shao W., Meng H. (2022). Performance and Application Evaluation of SARS-CoV-2 Antigen Assay. J. Med. Virol..

[11] Xu Z., Chen D., Li T., Yan J., Zhu J., He T., Hu R., Li Y., Yang Y., Liu M. (2022). Microfluidic Space Coding for Multiplexed Nucleic Acid Detection via CRISPR-Cas12a and Recombinase Polymerase Amplification. Nat. Commun..

[12] Jackson’ J.B., Balfour H.H. (1988). Practical Diagnostic Testing for Human Immunodeficiency Virus. Clin. Microbiol. Rev..

[13] Smirnov I., Shafer R.H. (2000). Effect of Loop Sequence and Size on DNA Aptamer Stability. Biochemistry.

[14] Bishop G.R., Ren J., Polander B.C., Jeanfreau B.D., Trent J.O., Chaires J.B. (2007). Energetic Basis of Molecular Recognition in a DNA Aptamer. Biophys. Chem..

[15] Xia T., Yuan J., Fang X. (2013). Conformational Dynamics of an ATP-Binding DNA Aptamer: A Single-Molecule Study. J. Phys. Chem. B.

[16] Tan L., Neoh K.G., Kang E.-T., Choe W.-S., Su X. (2012). Affinity Analysis of DNA Aptamer–Peptide Interactions Using Gold Nanoparticles. Anal. Biochem..

[17] Lai J.-C., Hong C.-Y. (2014). Magnetic-Assisted Rapid Aptamer Selection (MARAS) for Generating High-Affinity DNA Aptamer Using Rotating Magnetic Fields. ACS Comb. Sci..

[18] Minagawa H., Kataoka Y., Kuwahara M., Horii K., Shiratori I., Waga I. (2020). A High Affinity Modified DNA Aptamer Containing Base-Appended Bases for Human β-Defensin. Anal. Biochem..

[19] Xue C., Zhang S., Yu X., Hu S., Lu Y., Wu Z. (2020). Periodically Ordered, Nuclease-Resistant DNA Nanowires Decorated with Cell-Specific Aptamers as Selective Theranostic Agents. Angew. Chem. Int. Ed..

[20] Xie N., Liu S., Yang X., He X., Huang J., Wang K. (2017). DNA Tetrahedron Nanostructures for Biological Applications: Biosensors and Drug Delivery. Analyst.

[21] Sameiyan E., Bagheri E., Ramezani M., Alibolandi M., Abnous K., Taghdisi S.M. (2019). DNA Origami-Based Aptasensors. Biosens. Bioelectron..

[22] Zhang C., Chen J., Sun R., Huang Z., Luo Z., Zhou C., Wu M., Duan Y., Li Y. (2020). The Recent Development of Hybridization Chain Reaction Strategies in Biosensors. ACS Sens..

[23] Loretan M., Domljanovic I., Lakatos M., Rüegg C., Acuna G.P. (2020). DNA Origami as Emerging Technology for the Engineering of Fluorescent and Plasmonic-Based Biosensors. Materials.

[24] Yang H., Zhou Y., Liu J. (2020). G-Quadruplex DNA for Construction of Biosensors. TrAC Trends Anal. Chem..

[25] Wang S. (2021). Construction of DNA Biosensors for Mercury (II) Ion Detection Based on Enzyme-Driven Signal Amplification Strategy. Biomolecules.

[26] Wang F., Zhang Y., Lu M., Du Y., Chen M., Meng S., Ji W., Sun C. (2021). Near-Infrared Band Gold Nanoparticles-Au Film “Hot Spot” Model Based Label-Free Ultratrace Lead (II) Ions Detection via Fiber SPR DNAzyme Biosensor. Sens. Actuators B Chem..

[27] Zhai J., Cui H., Yang R. (1997). DNA Based Biosensors. Biotechnol. Adv..

[28] Wen Y., Liu G., Pei H., Li L., Xu Q., Liang W., Li Y., Xu L., Ren S. (2013). DNA Nanostructure-Based Ultrasensitive Electrochemical MicroRNA Biosensor. Methods.

[29] Li Y., Liu S., Deng Q. (2018). A Sensitive Colorimetric DNA Biosensor for Specific Detection of the HBV Gene Based on Silver-Coated Glass Slide and G-Quadruplex-Hemin DNAzyme. J. Med. Virol..

[30] Xie B., Qiu G., Hu P., Liang Z., Liang Y., Sun B., Bai L., Jiang Z., Chen J. (2018). Simultaneous Detection of Dengue and Zika Virus RNA Sequences with a Three-Dimensional Cu-Based Zwitterionic Metal–Organic Framework, Comparison of Single and Synchronous Fluorescence Analysis. Sens. Actuators B Chem..

[31] Chauhan N., Xiong Y., Ren S., Dwivedy A., Magazine N., Zhou L., Jin X., Zhang T., Cunningham B.T., Yao S. (2022). Net-Shaped DNA Nanostructures Designed for Rapid/Sensitive Detection and Potential Inhibition of the SARS-CoV-2 Virus. J. Am. Chem. Soc..

[32] Ito K., Hashimoto K., Ishimori Y. (1996). Quantitative Analysis for Solid-Phase Hybridization Reaction and Binding Reaction of DNA Binder to Hybrids Using a Quartz Crystal Microbalance. Anal. Chim. Acta.

[33] Fawcett N.C., Evans J.A., Chien L.-C., Flowers N. (1988). Nucleic Acid Hybridization Detected by Piezoelectric Resonance. Anal. Lett..

[34] Ye D., Zuo X., Fan C. (2018). DNA Nanotechnology-Enabled Interfacial Engineering for Biosensor Development. Annu. Rev. Anal. Chem..

[35] Shen L., Wang P., Ke Y. (2021). DNA Nanotechnology-Based Biosensors and Therapeutics. Adv. Healthc. Mater..

[36] Hua Y., Ma J., Li D., Wang R. (2022). DNA-Based Biosensors for the Biochemical Analysis: A Review. Biosensors.

[37] Seeman N.C. (1982). Nucleic Acid Junctions and Lattices. J. Theor. Biol..

[38] Ke Y., Ong L.L., Shih W.M., Yin P. (2012). Three-Dimensional Structures Self-Assembled from DNA Bricks. Science.

[39] Gerling T., Wagenbauer K.F., Neuner A.M., Dietz H. (2015). Dynamic DNA Devices and Assemblies Formed by Shape-Complementary, Non–Base Pairing 3D Components. Science.

[40] Rahbani J.F., Vengut-Climent E., Chidchob P., Gidi Y., Trinh T., Cosa G., Sleiman H.F. (2018). DNA Nanotubes with Hydrophobic Environments: Toward New Platforms for Guest Encapsulation and Cellular Delivery. Adv. Healthc. Mater..

[41] Zhou Y., Dong J., Zhou C., Wang Q. (2022). Finite Assembly of Three-Dimensional DNA Hierarchical Nanoarchitectures through Orthogonal and Directional Bonding. Angew. Chem. Int. Ed..

[42] Parsons M.F., Allan M.F., Li S., Shepherd T.R., Ratanalert S., Zhang K., Pullen K.M., Chiu W., Rouskin S., Bathe M. (2023). 3D RNA-Scaffolded Wireframe Origami. Nat. Commun..

[43] Huang K., Yang D., Tan Z., Chen S., Xiang Y., Mi Y., Mao C., Wei B. (2019). Self-Assembly of Wireframe DNA Nanostructures from Junction Motifs. Angew. Chem. Int. Ed..

[44] Hung A.M., Micheel C.M., Bozano L.D., Osterbur L.W., Wallraff G.M., Cha J.N. (2010). Large-Area Spatially Ordered Arrays of Gold Nanoparticles Directed by Lithographically Confined DNA Origami. Nat. Nanotechnol..

[45] He Y., Chen Y., Liu H., Ribbe A.E., Mao C. (2005). Self-Assembly of Hexagonal DNA Two-Dimensional (2D) Arrays. J. Am. Chem. Soc..

[46] He Y., Tian Y., Chen Y., Deng Z., Ribbe A.E., Mao C. (2005). Sequence Symmetry as a Tool for Designing DNA Nanostructures. Angew. Chem. Int. Ed..

[47] He Y., Tian Y., Ribbe A.E., Mao C. (2006). Highly Connected Two-Dimensional Crystals of DNA Six-Point-Stars. J. Am. Chem. Soc..

[48] Zhang C., Su M., He Y., Zhao X., Fang P.-A., Ribbe A.E., Jiang W., Mao C. (2008). Conformational Flexibility Facilitates Self-Assembly of Complex DNA Nanostructures. Proc. Natl. Acad. Sci. USA.

[49] He Y., Ye T., Su M., Zhang C., Ribbe A.E., Jiang W., Mao C. (2008). Hierarchical Self-Assembly of DNA into Symmetric Supramolecular Polyhedra. Nature.

[50] Zhang C., Ko S.H., Su M., Leng Y., Ribbe A.E., Jiang W., Mao C. (2009). Symmetry Controls the Face Geometry of DNA Polyhedra. J. Am. Chem. Soc..

[51] He Y., Su M., Fang P.-A., Zhang C., Ribbe E., Jiang W., Mao C. (2010). On the Chirality of Self-Assembled DNA Octahedra. Angew. Chem. Int. Ed..

[52] Zhang C., Wu W., Li X., Tian C., Qian H., Wang G., Jiang W., Mao C. (2012). Controlling the Chirality of DNA Nanocages. Angew. Chem. Int. Ed..

[53] Tian C., Li X., Liu Z., Jiang W., Wang G., Mao C. (2014). Directed Self-Assembly of DNA Tiles into Complex Nanocages. Angew. Chem. Int. Ed..

[54] Li Y., Tian C., Liu Z., Jiang W., Structural C.M. (2015). Transformation: Assembly of an Otherwise Inaccessible DNA Nanocage. Angew. Chem. Int. Ed..

[55] Wang P., Wu S., Tian C., Yu G., Jiang W., Wang G., Mao C. (2016). Retrosynthetic Analysis-Guided Breaking Tile Symmetry for the Assembly of Complex DNA Nanostructures. J. Am. Chem. Soc..

[56] Wu X., Wu C., Ding F., Tian C., Jiang W., Mao C., Zhang C. (2017). Binary Self-Assembly of Highly Symmetric DNA Nanocages via Sticky-End Engineering. Chin. Chem. Lett..

[57] Wang T., Bai T., Tan Z., Ohayon Y.P., Sha R., Vecchioni S., Seeman N.C., Wei B. (2023). Mesojunction-Based Design Paradigm of Structural DNA Nanotechnology. J. Am. Chem. Soc..

[58] Vecchioni S., Lu B., Janowski J., Woloszyn K., Jonoska N., Seeman N.C., Mao C., Ohayon Y.P., Sha R. (2023). The Rule of Thirds: Controlling Junction Chirality and Polarity in 3D DNA Tiles. Small.

[59] Lu B., Vecchioni S., Ohayon Y.P., Woloszyn K., Markus T., Mao C., Seeman N.C., Canary J.W., Sha R. (2023). Highly Symmetric, Self-Assembling 3D DNA Crystals with Cubic and Trigonal Lattices. Small.

[60] Duckett D.R., Murchie A.I.H., Diekmann S., von Kitzing E., Kemper B., Lilley D.M.J. (1988). The Structure of the Holliday Junction, and Its Resolution. Cell.

[61] Ma R.-I., Kallenbach N.R., Sheardy R.D., Petrillo M.L., Seeman N.C. (1986). Three-Arm Nucleic Acid Junctions Are Flexible. Nucleic Acids Res..

[62] Wang Y., Mueller J.E., Kemper B., Seeman N.C. (1991). Assembly and Characterization of Five-Arm and Six-Arm DNA Branched Junctions. Biochemistry.

[63] Wang X., Seeman N.C. (2007). Assembly and Characterization of 8-Arm and 12-Arm DNA Branched Junctions. J. Am. Chem. Soc..

[64] Fu T.J., Seeman N.C. (1993). DNA Double-Crossover Molecules. Biochemistry.

[65] LaBean T.H., Yan H., Kopatsch J., Liu F., Winfree E., Reif J.H., Seeman N.C. (2000). Construction, Analysis, Ligation, and Self-Assembly of DNA Triple Crossover Complexes. J. Am. Chem. Soc..

[66] Gu H., Chao J., Xiao S.-J., Seeman N.C. (2010). A Proximity-Based Programmable DNA Nanoscale Assembly Line. Nature.

[67] Mathieu F., Liao S., Kopatsch J., Wang T., Mao C., Seeman N.C. (2005). Six-Helix Bundles Designed from DNA. Nano Lett..

[68] Dong Y., Yao C., Zhu Y., Yang L., Luo D., Yang D. (2020). DNA Functional Materials Assembled from Branched DNA: Design, Synthesis, and Applications. Chem. Rev..

[69] Goodman R.P., Schaap I.A.T., Tardin C.F., Erben C.M., Berry R.M., Schmidt C.F., Turberfield A.J. (2005). Rapid Chiral Assembly of Rigid DNA Building Blocks for Molecular Nanofabrication. Science.

[70] Zhang Y., Seeman N.C. (1994). Construction of a DNA-Truncated Octahedron. J. Am. Chem. Soc..

[71] Hong F., Jiang S., Lan X., Narayanan R.P., Šulc P., Zhang F., Liu Y., Yan H. (2018). Layered-Crossover Tiles with Precisely Tunable Angles for 2D and 3D DNA Crystal Engineering. J. Am. Chem. Soc..

[72] Li S., Jiang Q., Liu S., Zhang Y., Tian Y., Song C., Wang J., Zou Y., Anderson G.J., Han J.-Y. (2018). A DNA Nanorobot Functions as a Cancer Therapeutic in Response to a Molecular Trigger In Vivo. Nat. Biotechnol..

[73] Tuerk C., Gold L. (1990). Systematic Evolution of Ligands by Exponential Enrichment: RNA Ligands to Bacteriophage T4 DNA Polymerase. Science.

[74] Ellington A.D., Szostak J.W. (1990). In Vitro Selection of RNA Molecules That Bind Specific Ligands. Nature.

[75] Jenison R.D., Gill S.C., Pardi A., Polisky B. (1994). High-Resolution Molecular Discrimination by RNA. Science.

[76] Stojanovic M.N., De Prada P., Landry D.W. (2001). Aptamer-Based Folding Fluorescent Sensor for Cocaine. J. Am. Chem. Soc..

[77] Liu J., Lu Y. (2006). Fast Colorimetric Sensing of Adenosine and Cocaine Based on a General Sensor Design Involving Aptamers and Nanoparticles. Angew. Chem. Int. Ed..

[78] Baker B.R., Lai R.Y., Wood M.S., Doctor E.H., Heeger A.J., Plaxco K.W. (2006). An Electronic, Aptamer-Based Small-Molecule Sensor for the Rapid, Label-Free Detection of Cocaine in Adulterated Samples and Biological Fluids. J. Am. Chem. Soc..

[79] Liu J., Cao Z., Lu Y. (2009). Functional Nucleic Acid Sensors. Chem. Rev..

[80] Santini J.T., Richards A.C., Scheidt R., Cima M.J., Langer R. (2000). Microchips as Controlled Drug-Delivery Devices. Angew. Chem. Int. Ed..

[81] Hamaguchi N., Ellington A., Stanton M. (2001). Aptamer Beacons for the Direct Detection of Proteins. Anal. Biochem..

[82] Yu H., Alkhamis O., Canoura J., Liu Y., Xiao Y. (2021). Advances and Challenges in Small-Molecule DNA Aptamer Isolation, Characterization, and Sensor Development. Angew. Chem. Int. Ed..

[83] Xi Z., Huang R., Li Z., He N., Wang T., Su E., Selection Y.D. (2015). of HBsAg-Specific DNA Aptamers Based on Carboxylated Magnetic Nanoparticles and Their Application in the Rapid and Simple Detection of Hepatitis B Virus Infection. ACS Appl. Mater. Interfaces.

[84] Gao H., Zhao J., Huang Y., Cheng X., Wang S., Han Y., Xiao Y., Lou X. (2019). Universal Design of Structure-Switching Aptamers with Signal Reporting Functionality. Anal. Chem..

[85] Han C., Li W., Li Q., Xing W., Luo H., Ji H., Fang X., Luo Z., Zhang L. (2022). CRISPR/Cas12a-Derived Electrochemical Aptasensor for Ultrasensitive Detection of COVID-19 Nucleocapsid Protein. Biosens. Bioelectron..

[86] Terracciano M., Rea I., Borbone N., Moretta R., Oliviero G., Piccialli G., De Stefano L. (2019). Silicon-Based Aptasensors: The Next Generation of Label-Free Devices for Health Monitoring. Molecules.

[87] Prante M., Segal E., Scheper T., Bahnemann J., Walter J. (2020). Aptasensors for Point-of-Care Detection of Small Molecules. Biosensors.

[88] Yamamoto R., Kumar P.K.R. (2000). Molecular Beacon Aptamer Fluoresces in the Presence of Tat Protein of HIV-1: Tat-Dependent Fluorescent Beacon Aptamer. Genes Cells.

[89] Tseng Y.-T., Wang C.-H., Chang C.-P., Lee G.-B. (2016). Integrated Microfluidic System for Rapid Detection of Influenza H1N1 Virus Using a Sandwich-Based Aptamer Assay. Biosens. Bioelectron..

[90] Wang C.-H., Chang C.-P., Lee G.-B. (2016). Integrated Microfluidic Device Using a Single Universal Aptamer to Detect Multiple Types of Influenza Viruses. Biosens. Bioelectron..

[91] Geng Z., Wang L., Liu K., Liu J., Tan W. (2021). Enhancing Anti-PD-1 Immunotherapy by Nanomicelles Self-Assembled from Multivalent Aptamer Drug Conjugates. Angew. Chem. Int. Ed..

[92] Schmitz A., Weber A., Bayin M., Breuers S., Fieberg V., Famulok M., Mayer G. (2021). A SARS-CoV-2 Spike Binding DNA Aptamer That Inhibits Pseudovirus Infection by an RBD-Independent Mechanism. Angew. Chem. Int. Ed..

[93] Yang M., Chen X., Zhu L., Lin S., Li C., Li X., Huang K., Xu W. (2021). Aptamer-Functionalized DNA–Silver Nanocluster Nanofilm for Visual Detection and Elimination of Bacteria. ACS Appl. Mater. Interfaces.

[94] Chen H., Park S.-K., Joung Y., Kang T., Lee M.-K., Choo J. (2022). SERS-Based Dual-Mode DNA Aptasensors for Rapid Classification of SARS-CoV-2 and Influenza A/H1N1 Infection. Sens. Actuators B Chem..

[95] Koonin E.V., Krupovic M., Agol V.I. (2021). The Baltimore Classification of Viruses 50 Years Later: How Does It Stand in the Light of Virus Evolution?. Microbiol. Mol. Biol. Rev..

[96] Rowe W.P., Huebner R.J., Gilmore L.K., Parrott R.O.H., Ward T.G. (1953). Isolation of a Cytopathogenic Agent from Human Adenoids Undergoing Spontaneous Degeneration in Tissue Culture. Exp. Biol. Med..

[97] Hllleman M.R., Werner J.H. (1954). Recovery of New Agent from Patients with Acute Respiratory Illness. Exp. Biol. Med..

[98] Kulanayake S., Tikoo S. (2021). Adenovirus Core Proteins: Structure and Function. Viruses.

[99] Gallardo J., Pérez-Illana M., Martín-González N., Martín C.S. (2021). Adenovirus Structure: What Is New?. Int. J. Mol. Sci..

[100] Lenman A., Liaci A.M., Liu Y., Frängsmyr L., Frank M., Blaum B.S., Chai W., Podgorski I.I., Harrach B., Benkő M. (2018). Polysialic Acid Is a Cellular Receptor for Human Adenovirus 52. Proc. Natl. Acad. Sci. USA.

[101] Badr K.R., Parente-Rocha J.A., Baeza L.C., Ficcadori F.S., Souza M., Soares C.M., Guissoni A.C.P., Almeida T.N., Cardoso D.D. (2019). Quantitative Proteomic Analysis of A549 Cells Infected with Human Adenovirus Type 2. J. Med. Virol..

[102] Mao S. (2019). The Fitness Landscape of AAV. Science.

[103] Wang X., Zhang F., Su R., Li X., Chen W., Chen Q., Yang T., Wang J., Liu H., Fang Q. (2018). Structure of RNA Polymerase Complex and Genome within a DsRNA Virus Provides Insights into the Mechanisms of Transcription and Assembly. Proc. Natl. Acad. Sci. USA.

[104] Inoue-Nagata A.K., Jordan R., Kreuze J., Li F., López-Moya J.J., Mäkinen K., Ohshima K., Wylie S.J. (2022). ICTV Report Consortium. ICTV Virus Taxonomy Profile: Potyviridae 2022: This Article Is Part of the ICTV Virus Taxonomy Profiles Collection. J. Gen. Virol..

[105] Jackson C.B., Farzan M., Chen B., Choe H. (2022). Mechanisms of SARS-CoV-2 Entry into Cells. Nat. Rev. Mol. Cell Biol..

[106] Wrapp D., Wang N., Corbett K.S., Goldsmith J.A., Hsieh C.-L., Abiona O., Graham B.S., McLellan J.S. (2020). Cryo-EM Structure of the 2019-NCoV Spike in the Prefusion Conformation. Science.

[107] Yan R., Zhang Y., Li Y., Xia L., Guo Y., Zhou Q. (2020). Structural Basis for the Recognition of SARS-CoV-2 by Full-Length Human ACE2. Science.

[108] Xue K.S., Moncla L.H., Bedford T., Bloom J.D. (2018). Within-Host Evolution of Human Influenza Virus. Trends Microbiol..

[109] Ye Q., Krug R.M., Tao Y.J. (2006). The Mechanism by Which Influenza A Virus Nucleoprotein Forms Oligomers and Binds RNA. Nature.

[110] Obayashi E., Yoshida H., Kawai F., Shibayama N., Kawaguchi A., Nagata K., Tame J.R.H., Park S.-Y. (2008). The Structural Basis for an Essential Subunit Interaction in Influenza Virus RNA Polymerase. Nature.

[111] Sugiyama K., Obayashi E., Kawaguchi A., Suzuki Y., Tame J.R.H., Nagata K., Park S.-Y. (2009). Structural Insight into the Essential PB1–PB2 Subunit Contact of the Influenza Virus RNA Polymerase. EMBO J..

[112] Wandzik J.M., Kouba T., Karuppasamy M., Pflug A., Drncova P., Provaznik J., Azevedo N., Cusack S. (2020). A Structure-Based Model for the Complete Transcription Cycle of Influenza Polymerase. Cell.

[113] Ma Y., He Z., Tan T., Li W., Zhang Z., Song S., Zhang X., Hu Q., Zhou P., Wu Y. (2016). Real-Time Imaging of Single HIV-1 Disassembly with Multicolor Viral Particles. ACS Nano.

[114] Chen Y., Dierich M.P. (1998). Biological Function of H1V-1 Transmembrane Protein Gp41: A Study on a Putative Cellular Receptor of Gp41. Chin. Sci. Bull..

[115] Chen J.Y., Feeney E.R., Chung R.T. (2014). HCV and HIV Co-Infection: Mechanisms and Management. Nat. Rev. Gastroenterol. Hepatol..

[116] Galibert F., Mandart E., Fitoussi F., Tiollais P., Charnay P. (1979). Nucleotide Sequence of the Hepatitis B Virus Genome (Subtype Ayw) Cloned in *E. Coli*. Nature.

[117] Kay A., Zoulim F. (2007). Hepatitis B Virus Genetic Variability and Evolution. Virus Res..

[118] Kostyusheva A., Brezgin S., Glebe D., Kostyushev D., Chulanov V. (2021). Host-Cell Interactions in HBV Infection and Pathogenesis: The Emerging Role of M6A Modification. Emerg. Microbes Infect..

[119] Steinhardt E., Israeli C., Lambert R.A. (1913). Studies on the Cultivation of the Virus of Vaccinia. J. Infect. Dis..

[120] Ziegler C.G.K., Allon S.J., Nyquist S.K., Mbano I.M., Miao V.N., Tzouanas C.N., Cao Y., Yousif A.S., Bals J., Hauser B.M. (2020). SARS-CoV-2 Receptor ACE2 Is an Interferon-Stimulated Gene in Human Airway Epithelial Cells and Is Detected in Specific Cell Subsets across Tissues. Cell.

[121] Feng R., Wan K., Sui X., Zhao N., Li H., Lei W., Yu J., Liu X., Shi X., Zhai M. (2021). Anchoring Single Pt Atoms and Black Phosphorene Dual Co-Catalysts on CdS Nanospheres to Boost Visible-Light Photocatalytic H2 Evolution. Nano Today.

[122] Bidwell D.E., Malaria A.V. (1981). Diagnosis by Enzyme-Linked Immunosorbent Assays. BMJ.

[123] Lequin R.M. (2005). Enzyme Immunoassay (EIA)/Enzyme-Linked Immunosorbent Assay (ELISA). Clin. Chem..

[124] Faulk W.P., Taylor G.M. (1971). Communication to the Editors. Immunochemistry.

[125] Spielberg F., Ryder R., Harris J., Heyward W., Kabeya C., Kifuani N.K., Bender T., Quinn T. (1989). Filed Testing and Comparative Evaluation of Rapid, Visually Read Screening Assays for Antibody to Human Immunodeficiency Virus. Lancet.

[126] Xu M., Lu F., Lyu C., Wu Q., Zhang J., Tian P., Xue L., Xu T., Wang D. (2021). Broad-Range and Effective Detection of Human Noroviruses by Colloidal Gold Immunochromatographic Assay Based on the Shell Domain of the Major Capsid Protein. BMC Microbiol..

[127] Peng P., Liu C., Li Z., Xue Z., Mao P., Hu J., Xu F., Yao C., You M. (2022). Emerging ELISA Derived Technologies for in Vitro Diagnostics. TrAC Trends Anal. Chem..

[128] Ho S.N., Hunt H.D., Horton R.M., Pullen J.K., Pease L.R. (1989). Site-Directed Mutagenesis by Overlap Extension Using the Polymerase Chain Reaction. Gene.

[129] Tan I.L., Dimamay M.P.S., Buerano C.C., Alfon J.A.R., Tanig C.Z., Matias R.R., Natividad F.F. (2010). Development and Evaluation of a Fluorogenic Real-Time RT-PCR for the Detection of Dengue 3 Virus. J. Med. Virol..

[130] Pham J., Meyer S., Nguyen C., Williams A., Hunsicker M., McHardy I., Gendlina I., Goldstein D.Y., Fox A.S., Hudson A. (2020). Performance Characteristics of a High-Throughput Automated Transcription-Mediated Amplification Test for SARS-CoV-2 Detection. J. Clin. Microbiol..

[131] Fowler V.L., Armson B., Gonzales J.L., Wise E.L., Howson E.L.A., Vincent-Mistiaen Z., Fouch S., Maltby C.J., Grippon S., Munro S. (2021). A Highly Effective Reverse-Transcription Loop-Mediated Isothermal Amplification (RT-LAMP) Assay for the Rapid Detection of SARS-CoV-2 Infection. J. Infect..

[132] Li J., Zhou J., Xia Y., Rui Y., Yang X., Xie G., Jiang G., Wang H. (2023). Rolling Circle Extension-Assisted Loop-Mediated Isothermal Amplification (Rol-LAMP) Method for Locus-Specific and Visible Detection of RNA N6-Methyladenosine. Nucleic Acids Res..

[133] Glökler J., Lim T.S., Ida J., Frohme M. (2021). Isothermal Amplifications—A Comprehensive Review on Current Methods. Crit. Rev. Biochem. Mol. Biol..

[134] Ruijter J.M., Barnewall R.J., Marsh I.B., Szentirmay A.N., Quinn J.C., Van Houdt R., Gunst Q.D., Van Den Hoff M.J.B. (2021). Efficiency Correction Is Required for Accurate Quantitative PCR Analysis and Reporting. Clin. Chem..

[135] Liu Y., Kumar S., Taylor R.E. (2018). Mix-and-match Nanobiosensor Design: Logical and Spatial Programming of Biosensors Using Self-assembled DNA Nanostructures. Wiley Interdiscip. Rev. Nanomed. Nanobiotechnol..

[136] Fan S., Cheng J., Liu Y., Wang D., Luo T., Dai B., Zhang C., Cui D., Ke Y., Song J. (2020). Proximity-Induced Pattern Operations in Reconfigurable DNA Origami Domino Array. J. Am. Chem. Soc..

[137] Terracciano M., De Stefano L., Borbone N., Politi J., Oliviero G., Nici F., Casalino M., Piccialli G., Dardano P., Varra M. (2016). Solid Phase Synthesis of a Thrombin Binding Aptamer on Macroporous Silica for Label Free Optical Quantification of Thrombin. RSC Adv..

[138] Eivazzadeh-Keihan R., Pashazadeh-Panahi P., Baradaran B., de la Guardia M., Hejazi M., Sohrabi H., Mokhtarzadeh A., Maleki A. (2018). Recent Progress in Optical and Electrochemical Biosensors for Sensing of Clostridium Botulinum Neurotoxin. TrAC Trends Anal. Chem..

[139] Tong L., Xu H., Käll M. (2014). Nanogaps for SERS Applications. MRS Bull..

[140] Song C., Jiang X., Yang Y., Zhang J., Larson S., Zhao Y., Wang L. (2020). High-Sensitive Assay of Nucleic Acid Using Tetrahedral DNA Probes and DNA Concatamers with a Surface-Enhanced Raman Scattering/Surface Plasmon Resonance Dual-Mode Biosensor Based on a Silver Nanorod-Covered Silver Nanohole Array. ACS Appl. Mater. Interfaces.

[141] Khalil I., Yehye W.A., Julkapli N.M., Rahmati S., Sina A.A.I., Basirun W.J., Johan M.R. (2019). Graphene Oxide and Gold Nanoparticle Based Dual Platform with Short DNA Probe for the PCR Free DNA Biosensing Using Surface-Enhanced Raman Scattering. Biosens. Bioelectron..

[142] Das G.M., Managò S., Mangini M., De Luca A.C. (2021). Biosensing Using SERS Active Gold Nanostructures. Nanomaterials.

[143] Li Q., Huo H., Wu Y., Chen L., Su L., Zhang X., Song J., Yang H. (2023). Design and Synthesis of SERS Materials for In Vivo Molecular Imaging and Biosensing. Adv. Sci..

[144] Seymour E., Kanik F.E., Gür S.D., Bakhshpour-Yucel M., Araz A., Ünlü N.L., Ünlü M.S. (2023). Solid-Phase Optical Sensing Techniques for Sensitive Virus Detection. Sensors.

[145] Xia J., Li W., Sun M., Wang H. (2022). Application of SERS in the Detection of Fungi, Bacteria and Viruses. Nanomaterials.

[146] Mousavi S.M., Hashemi S.A., Rahmanian V., Kalashgrani M.Y., Gholami A., Omidifar N., Chiang W.-H. (2022). Highly Sensitive Flexible SERS-Based Sensing Platform for Detection of COVID-19. Biosensors.

[147] Saviñon-Flores F., Méndez E., López-Castaños M., Carabarin-Lima A., López-Castaños K.A., González-Fuentes M.A., Méndez-Albores A. (2021). A Review on SERS-Based Detection of Human Virus Infections: Influenza and Coronavirus. Biosensors.

[148] Song C., Zhang J., Liu Y., Guo X., Guo Y., Jiang X., Wang L. (2020). Highly Sensitive SERS Assay of DENV Gene via a Cascade Signal Amplification Strategy of Localized Catalytic Hairpin Assembly and Hybridization Chain Reaction. Sens. Actuators B Chem..

[149] Huh Y.S., Chung A.J., Cordovez B., Erickson D. (2009). Enhanced On-Chip SERS Based Biomolecular Detection Using Electrokinetically Active Microwells. Lab Chip.

[150] Park K.S., Choi A., Kim H.J., Park I., Eom M.-S., Yeo S.-G., Son R.G., Park T.-I., Lee G., Soh H.T. (2023). Ultra-Sensitive Label-Free SERS Biosensor with High-Throughput Screened DNA Aptamer for Universal Detection of SARS-CoV-2 Variants from Clinical Samples. Biosens. Bioelectron..

[151] Fen Y.W., Yunus W.M.M., Yusof N.A., Ishak N.S., Omar N.A.S., Zainudin A.A. (2015). Preparation, Characterization and Optical Properties of Ionophore Doped Chitosan Biopolymer Thin Film and Its Potential Application for Sensing Metal Ion. Optik.

[152] Zainudin A.A., Fen Y.W., Yusof N.A., Omar N.A.S. (2017). Structural, Optical and Sensing Properties of Ionophore Doped Graphene Based Bionanocomposite Thin Film. Optik.

[153] Omar N.A.S., Fen Y.W. (2018). Recent Development of SPR Spectroscopy as Potential Method for Diagnosis of Dengue Virus E-Protein. Sens. Rev..

[154] Murali S., Rustandi R., Zheng X., Payne A., Shang L. (2022). Applications of Surface Plasmon Resonance and Biolayer Interferometry for Virus–Ligand Binding. Viruses.

[155] Takemura K. (2021). Surface Plasmon Resonance (SPR)- and Localized SPR (LSPR)-Based Virus Sensing Systems: Optical Vibration of Nano- and Micro-Metallic Materials for the Development of Next-Generation Virus Detection Technology. Biosensors.

[156] Hassan M.M., Sium F.S., Islam F., Choudhury S.M. (2021). A Review on Plasmonic and Metamaterial Based Biosensing Platforms for Virus Detection. Sens. Bio-Sens. Res..

[157] Pandey P.S., Raghuwanshi S.K., Shadab A., Ansari M.T.I., Tiwari U.K., Kumar S. (2022). SPR Based Biosensing Chip for COVID-19 Diagnosis—A Review. IEEE Sens. J..

[158] Diao W., Tang M., Ding S., Li X., Cheng W., Mo F., Yan X., Ma H., Yan Y. (2018). Highly Sensitive Surface Plasmon Resonance Biosensor for the Detection of HIV-Related DNA Based on Dynamic and Structural DNA Nanodevices. Biosens. Bioelectron..

[159] Lee T., Kim G.H., Kim S.M., Hong K., Kim Y., Park C., Sohn H., Min J. (2019). Label-Free Localized Surface Plasmon Resonance Biosensor Composed of Multi-Functional DNA 3 Way Junction on Hollow Au Spike-like Nanoparticles (HAuSN) for Avian Influenza Virus Detection. Colloids Surf. B Biointerfaces.

[160] Chowdhury A.D., Takemura K., Khorish I.M., Nasrin F., Tun M.M.N., Morita K., Park E.Y. (2020). The Detection and Identification of Dengue Virus Serotypes with Quantum Dot and AuNP Regulated Localized Surface Plasmon Resonance. Nanoscale Adv..

[161] Oliveira N., Souza E., Ferreira D., Zanforlin D., Bezerra W., Borba M., Arruda M., Lopes K., Nascimento G., Martins D. (2015). A Sensitive and Selective Label-Free Electrochemical DNA Biosensor for the Detection of Specific Dengue Virus Serotype 3 Sequences. Sensors.

[162] Damborský P., Švitel J., Katrlík J. (2016). Optical Biosensors. Essays Biochem..

[163] Celiker T., Ghorbanizamani F., Moulahoum H., Celik E.G., Tok K., Zihnioglu F., Cicek C., Sertoz R., Arda B., Goksel T. (2022). Fluorescent Bioassay for SARS-CoV-2 Detection Using Polypyrene-g-Poly(ε-Caprolactone) Prepared by Simultaneous Photoinduced Step-Growth and Ring-Opening Polymerizations. Microchim. Acta.

[164] Sharma A., Mishra R.K., Goud K.Y., Mohamed M.A., Kummari S., Tiwari S., Li Z., Narayan R., Stanciu L.A., Marty J.L. (2021). Optical Biosensors for Diagnostics of Infectious Viral Disease: A Recent Update. Diagnostics.

[165] Kulzer F., Orrit M. (2004). Single-Molecule Optics. Annu. Rev. Phys. Chem..

[166] Shirani M., Kalantari H., Khodayar M.J., Kouchak M., Rahbar N. (2020). A Novel Strategy for Detection of Small Molecules Based on Aptamer/Gold Nanoparticles/Graphitic Carbon Nitride Nanosheets as Fluorescent Biosensor. Talanta.

[167] Salama A.M., Yasin G., Zourob M., Lu J. (2022). Fluorescent Biosensors for the Detection of Viruses Using Graphene and Two-Dimensional Carbon Nanomaterials. Biosensors.

[168] Ekiz-Kanik F., Sevenler D.D., Ünlü N.L., Chiari M., Ünlü M.S. (2017). Surface Chemistry and Morphology in Single Particle Optical Imaging. Nanophotonics.

[169] Sharma A., Khan R., Catanante G., Sherazi T., Bhand S., Hayat A., Marty J. (2018). Designed Strategies for Fluorescence-Based Biosensors for the Detection of Mycotoxins. Toxins.

[170] Maddali H., Miles C.E., Kohn J., O’Carroll D.M. (2021). Optical Biosensors for Virus Detection: Prospects for SARS-CoV-2/COVID-19. ChemBioChem.

[171] Shen W., Gao Z. (2015). Quantum Dots and Duplex-Specific Nuclease Enabled Ultrasensitive Detection and Serotyping of Dengue Viruses in One Step in a Single Tube. Biosens. Bioelectron..

[172] Wu P., Yan X.-P. (2013). Doped Quantum Dots for Chemo/Biosensing and Bioimaging. Chem. Soc. Rev..

[173] Teengam P., Nisab N., Chuaypen N., Tangkijvanich P., Vilaivan T., Chailapakul O. (2021). Fluorescent Paper-Based DNA Sensor Using Pyrrolidinyl Peptide Nucleic Acids for Hepatitis C Virus Detection. Biosens. Bioelectron..

[174] Jiao J., Duan C., Xue L., Liu Y., Sun W., Xiang Y. (2020). DNA Nanoscaffold-Based SARS-CoV-2 Detection for COVID-19 Diagnosis. Biosens. Bioelectron..

[175] Kwon P.S., Ren S., Kwon S.-J., Kizer M.E., Kuo L., Xie M., Zhu D., Zhou F., Zhang F., Kim D. (2020). Designer DNA Architecture Offers Precise and Multivalent Spatial Pattern-Recognition for Viral Sensing and Inhibition. Nat. Chem..

[176] Ochmann S.E., Vietz C., Trofymchuk K., Acuna G.P., Lalkens B., Tinnefeld P. (2017). Optical Nanoantenna for Single Molecule-Based Detection of Zika Virus Nucleic Acids without Molecular Multiplication. Anal. Chem..

[177] Chowdhury A.D., Ganganboina A.B., Nasrin F., Takemura K., Doong R.-A., Utomo D.I.S., Lee J., Khoris I.M., Park E.Y. (2018). Femtomolar Detection of Dengue Virus DNA with Serotype Identification Ability. Anal. Chem..

[178] Gogianu L., Popescu M.C., Vasile B.S., Mihalache I., Anghel E.M., Damian C.M., Salceanu A., Boldeiu A., Constantin E., Radoi A. (2023). Microarray Biochip Fabricated on Silicon Nanowires/Carbon Dots Heterostructures for Enhanced Viral DNA Detection. Appl. Surf. Sci..

[179] Li S., Li C., Wang Y., Li H., Xia F. (2020). Re-Engineering Electrochemical Aptamer-Based Biosensors to Tune Their Useful Dynamic Range via Distal-Site Mutation and Allosteric Inhibition. Anal. Chem..

[180] Huang H., Bai W., Dong C., Guo R., Liu Z. (2015). An Ultrasensitive Electrochemical DNA Biosensor Based on Graphene/Au Nanorod/Polythionine for Human Papillomavirus DNA Detection. Biosens. Bioelectron..

[181] Vermisoglou E., Panáček D., Jayaramulu K., Pykal M., Frébort I., Kolář M., Hajdúch M., Zbořil R., Otyepka M. (2020). Human Virus Detection with Graphene-Based Materials. Biosens. Bioelectron..

[182] Kurzątkowska K., Sirko A., Zagórski-Ostoja W., Dehaen W., Radecka H., Radecki J. (2015). Electrochemical Label-Free and Reagentless Genosensor Based on an Ion Barrier Switch-off System for DNA Sequence-Specific Detection of the Avian Influenza Virus. Anal. Chem..

[183] Li C., Sun L., Xu Z., Wu X., Liang T., Shi W. (2020). Experimental Investigation and Error Analysis of High Precision FBG Displacement Sensor for Structural Health Monitoring. Int. J. Struct. Stab. Dyn..

[184] Kianfar E., Salimi M., Koohestani B. (2020). Methanol to Gasoline Conversion over CuO/ZSM-5 Catalyst Synthesized and Influence of Water on Conversion. Fine Chem. Eng..

[185] Kianfar E. (2020). An Experimental Study PVDF and PSF Hollow Fiber Membranes for Chemical Absorption Carbon Dioxide. Fine Chem. Eng..

[186] Lee T., Park S.Y., Jang H., Kim G.-H., Lee Y., Park C., Mohammadniaei M., Lee M.-H., Min J. (2019). Fabrication of Electrochemical Biosensor Consisted of Multi-Functional DNA Structure/Porous Au Nanoparticle for Avian Influenza Virus (H5N1) in Chicken Serum. Mater. Sci. Eng. C.

[187] Fu J., Wu J., Zhang R., Wu Q., Ju H. (2021). Electrochemical Biosensing of DENV Nucleic Acid Amplified with Triplet Nanostructure-Mediated Dendritic Hybridization Chain Reaction. Sens. Actuators B Chem..

[188] Dong S., Zhao R., Zhu J., Lu X., Li Y., Qiu S., Jia L., Jiao X., Song S., Fan C. (2015). Electrochemical DNA Biosensor Based on a Tetrahedral Nanostructure Probe for the Detection of Avian Influenza A (H7N9) Virus. ACS Appl. Mater. Interfaces.

[189] Mahmoodi P., Rezayi M., Rasouli E., Avan A., Gholami M., Mobarhan M.G., Karimi E., Alias Y. (2020). Early-Stage Cervical Cancer Diagnosis Based on an Ultra-Sensitive Electrochemical DNA Nanobiosensor for HPV-18 Detection in Real Samples. J. Nanobiotechnol..

[190] Weng X., Li C., Chen C., Wang G., Xia C., Zheng L. (2023). A Microfluidic Device for Tobacco Ringspot Virus Detection by Electrochemical Impedance Spectroscopy. Micromachines.

[191] Chen Y.-S., Huang C.-H., Pai P.-C., Seo J., Lei K.F. (2023). A Review on Microfluidics-Based Impedance Biosensors. Biosensors.

[192] Wang S., Li L., Jin H., Yang T., Bao W., Huang S., Wang J. (2013). Electrochemical Detection of Hepatitis B and Papilloma Virus DNAs Using SWCNT Array Coated with Gold Nanoparticles. Biosens. Bioelectron..

[193] Štukovnik Z., Bren U. (2022). Recent Developments in Electrochemical-Impedimetric Biosensors for Virus Detection. Int. J. Mol. Sci..

[194] Shariati M. (2021). Impedimetric Biosensor for Monitoring Complementary DNA from Hepatitis B Virus Based on Gold Nanocrystals. J. Electrochem. Soc..

[195] Steinmetz M., Lima D., Viana A.G., Fujiwara S.T., Pessôa C.A., Etto R.M., Wohnrath K. (2019). A Sensitive Label-Free Impedimetric DNA Biosensor Based on Silsesquioxane-Functionalized Gold Nanoparticles for Zika Virus Detection. Biosens. Bioelectron..

[196] Ilkhani H., Farhad S. (2018). A Novel Electrochemical DNA Biosensor for Ebola Virus Detection. Anal. Biochem..

[197] Shariati M., Ghorbani M., Sasanpour P., Karimizefreh A. (2019). An Ultrasensitive Label Free Human Papilloma Virus DNA Biosensor Using Gold Nanotubes Based on Nanoporous Polycarbonate in Electrical Alignment. Anal. Chim. Acta.

[198] Faria H.A.M., Zucolotto V. (2019). Label-Free Electrochemical DNA Biosensor for Zika Virus Identification. Biosens. Bioelectron..

[199] Singhal C., Pundir C.S., Narang J. (2017). A Genosensor for Detection of Consensus DNA Sequence of Dengue Virus Using ZnO/Pt-Pd Nanocomposites. Biosens. Bioelectron..

[200] Bhardwaj J., Chaudhary N., Kim H., Jang J. (2019). Subtyping of Influenza A H1N1 Virus Using a Label-Free Electrochemical Biosensor Based on the DNA Aptamer Targeting the Stem Region of HA Protein. Anal. Chim. Acta.

[201] Ding H., Li J., Chen N., Hu X., Yang X., Guo L., Li Q., Zuo X., Wang L., Ma Y. (2018). DNA Nanostructure-Programmed Like-Charge Attraction at the Cell-Membrane Interface. ACS Cent. Sci..

[202] Praetorius F., Kick B., Behler K.L., Honemann M.N., Weuster-Botz D., Dietz H. (2017). Biotechnological Mass Production of DNA Origami. Nature.

[203] Huo S., Li H., Boersma A.J., Herrmann A. (2019). DNA Nanotechnology Enters Cell Membranes. Adv. Sci..

